# Involvement of the ovarian-specific Mro-IR in oogonia differentiation and oocyte development in freshwater giant prawn *Macrobrachium rosenbergii*

**DOI:** 10.3389/fendo.2025.1516849

**Published:** 2025-04-03

**Authors:** Wen-Ming Ma, Hai-Jing Xu

**Affiliations:** College of Advanced Agricultural Sciences, Zhejiang Wanli University, Ningbo, Zhejiang, China

**Keywords:** Mro-IR, gene knockdown, oocyte proliferation, ovarian development, insulin-like receptor

## Abstract

**Introduction:**

Mro-IR is an insulin-like receptor and is uniquely expressed in the ovary of the freshwater giant prawn *Macrobrachium rosenbergii*. However, the understanding of this conserved receptor involved in the molecular mechanism underpinning ovarian development and female reproduction of *M. rosenbergii* is still fragmentary.

**Methods:**

In the present study, *in vivo* knockdown of Mro-IR in the proliferative stage and premature stage of ovarian development in female prawn induced abnormal oogonia differentiation and disordered oocyte development.

**Results:**

The histological analysis showed that Mro-IR-silencing caused abnormal cellular morphology of some early vitellogenic oocytes (Oc2) and significantly delayed the proliferation of late vitellogenic oocytes (Oc3) in the proliferative stage of the ovary. Meanwhile, the Mro-IR silence led to the abnormal Oc3 with indistinct boundary and destructive structure of yolk accumulation in Oc3 in the premature stage of the ovary. Furthermore, to expound the potential roles of Mro-IR in ovarian development, a large amount of new data on significantly differentially upregulated and downregulated transcriptions was enriched, and the response of the primary Kyoto Encyclopedia of Genes and Genomes (KEGG) biological pathways was investigated. Their possible molecular regulatory relationships in gonad development and reproduction were briefly illustrated in the putative intuitive cascade regulation axis or networks.

**Discussion:**

This finding offered new insight regarding the mechanism of the IR gene family in ovarian development and reproduction of crustaceans.

## Introduction

Insulin signaling regulates various aspects of physiology, such as growth, longevity, aging, and reproduction. Insulin signaling acts cooperatively with gonadotropins to mediate various aspects of ovarian development in mammals and lower vertebrates (1). In humans, insulin signaling is crucial for female reproductive health. Meanwhile, insulin signaling directly regulates oocyte growth and maturation in *Drosophila melanogaster* and *Caenorhabditis elegans* (2). It is proposed that insulin signaling constitutes a key and evolutionarily ancient regulator of female reproduction.

Differently, the insulin-like signaling pathway is primarily focused on its significant roles in masculinization and male reproduction in crustaceans (3). Several male gonad-specific insulin-like peptides, commonly referred to as insulin-like androgenic gland hormones (IAG), have been identified, along with their receptors which have also been characterized. For example, in the red-claw crayfish, the insulin-like AG factor of *Cherax quadricarinatu*s (Cq-IAG) was expressed specifically in the male AG gland (4). Cq-IAG silencing led to testis degeneration and oocyte formation in male individuals in *C. quadricarinatus* (4). For another instance, Mr-IAG is a gender-specific insulin-like gene expressed in the AG in *M.rosenbergii* (5). Mr-IAG silencing led to the arrest of testicular spermatogenesis and spermatophore development, and male individuals with Mr-IAG knockdown obtained sex reversal into neo-females with female function and the capacity of all-males offspring reproduction (6, 7).

The insulin receptor (IR) is a transmembrane receptor that belongs to the ancient receptor tyrosine kinase superfamily (8, 9). The binding of the insulin-like peptide ligand initiates a cascade of phosphorylation events, stimulating the downstream signal transduction and resulting in cellular effect (8, 9). In recent years, some IAG receptors also have been identified in crustaceans, such as FcIAGR in the Chinese shrimp *Fenneropenaeus chinensis* (10), SvTKIR in Eastern rock lobster *Sagmariasus verreauxi* (11), and Mr-IR in the freshwater giant prawn *M.rosenbergii* (12). Mr-IR is the receptor of Mr-IAG and participates in the male sexual differentiation and the male sexual characteristics maintaining (12, 13). Mr-IR silencing resulted in AG hypertrophy and increased production of associated Mr-IAG, and an unusual abundance of immature sperm cells was seen in the distal sperm duct (12). Meanwhile, Mr-IR knockdown in the post-larval individuals efficiently retarded the spermatogenesis in the testis and induced sex reversal in the freshwater prawn *M. rosenbergii* (12, 13). These studies have extensively demonstrated that the insulin-like signaling pathway plays a significant role in the male sexual differentiation of crustaceans.

It is noteworthy that Mro-IR is another identified insulin-like receptor in *M. rosenbergii* and is uniquely expressed in the ovary (14). The expression of Mro-IR gradually increased with ovarian development and was closely correlated with the process of ovarian maturation (14). Exploring the molecular mechanism of Mro-IR involved in ovarian development and female reproduction would be highly significant. Ovarian development in *M. rosenbergii* follows a periodic maturation circle with notable changes, with cell proliferation and vitellogenesis being key events in this process. The proliferative stage and premature stage are the important stages for cell proliferation, vitellogenesis, and yolk accumulation during ovarian maturation (15, 16). Therefore, investigating the potential role of Mro-IR in ovarian development is a worthwhile subject for further research.

In the present study, the RNAi-mediated Mro-IR knockdown was carried out in the proliferative stage and premature stage of ovarian development in female prawns, respectively. The histological observation of ovarian development and potential effects of related-gene transcriptions in Mro-IR silenced females were synchronously analyzed by tissue section technique and comparing transcriptome sequencing, respectively. The possible molecular mechanisms of Mro-IR involved in the ovarian development and female reproduction of *M. rosenbergii* were expounded.

## 2 Materials and methods

### 2.1 Animals

The adult freshwater prawn *M. rosenbergii* was collected from Ningbo Yonggang Aquatic Seedling Technology Co., Ltd in Zhejiang, China, and acclimated in laboratory tanks one week before injection. Female prawns with 9.12 ± 1.05 cm body length (BL) and 20.03 ± 1.52 g body weight (BW), which had been cultured for eight months, were selected according to the female sexual appearance characteristics (one pair of the female genital pores located at the coxopodite of the third pereiopods) for the treatment. The body length of *M. rosenbergii* was measured as a straight line from the base of the eyestalk to the end of the telson. The injected prawns were reared in separated tanks (100 L) under a flow-through of freshwater system. The water was kept at a temperature of 27 ± 2°C, with dissolved oxygen above 5mg/L and a photoperiod of 14:10 (light: dark per day). Prawns were fed with artificial foods twice daily.

### 2.2 *In vivo* knockdown of Mro-IR by RNAi in female prawn

#### 2.2.1 Preparation of Mro-IR dsRNA

A pair of primers, RNAiF 5’-GCTCTAGAGGCAAGTGCGGCAGTAGGT-3’ and RNAiR 5’-CGGGATCCGGGTGTCCCAGTCCCATA-3’ was designed for the preparation of Mro-IR dsRNA (GenBank accession number OP966788.1). A 523-bp cDNA fragment was subcloned into the plasmid pET-T7 vector at the XbaI and BamHI restriction sites. The recombinant plasmids were transformed into Escherichia coli HT115. For a negative control, a 359-bp GFP dsRNA (GenBank accession number X83959.1) was designed and prepared. For RNAi experiments, the dsRNAs of Mro-IR and GFP were produced and purified as described (17).

#### 2.2.2 *In vivo* knockdown of Mro-IR by RNAi

The ovarian cycle of *M. rosenbergii* is generally classified into five stages, stage I of the rudimentary and spent ovary, stage II of the proliferative ovary, stage III of the premature ovary, stage IV of the mature ovary, and stage V of the oocyte-releasing ovary, respectively (15, 16). The female prawns during the proliferative stage and the premature stage of ovarian maturation were selected for the experiment by observing the appearances of coloration and the relative volume/morphology of ovaries from the dorsum, respectively. In the present study, females with a BL of 9.20 ± 0.20 cm and a BW of 18.43 ± 0.44 g in the GFP RNAi group, and a BL of 9.33 ± 0.15 cm and a BW 17.89 ± 0.46 g in the Mro-IR RNAi group were collected for gene knockdown during the proliferative stage of ovarian development. Meanwhile, the females with BL 9.00 ± 0.62 cm and BW 18.66 ± 0.82 g in the GFP RNAi group, and BL 9.50 ± 0.50 cm and BW 20.20 ± 1.31 g in the Mro-IR RNAi group were used for gene knockdown during the premature stage of ovarian development. 10μg dsRNA of Mro-IR in 0.9% (w/v) physiological saline was injected into each female prawn (N=10) through the arthrodial membrane at the base of the fifth pereiopods using a microinjection needle (glass replacement 3.5 nanoltr, item no. 4878, World Precision Instruments (WPI), Inc., 175 Sarasota Center Boulevard Sarasota, FL 34240-9258 USA). The control group (N=10) received an equal amount of GFP dsRNA injection. The injection was performed every five days during the animal experiment and two injections for 10 days were performed for each prawn. The value of the gonado-somatic index (GSI) was calculated as the ratio of gonad weight to body weight at the end of the animal experiment. The GSI values in the proliferative stage of females were 0.27 ± 0.04% in the GFP RNAi group and 0.35 ± 0.04% in the Mro-IR RNAi group, respectively. Meanwhile, the values of GSI in the premature stage of females were 2.17 ± 0.95% in the GFP RNAi group, and GSI 2.53 ± 0.83% in the Mro-IR RNAi group, respectively.

#### 2.2.3 The RNAi efficiency of Mro-IR silencing

To evaluate the RNAi efficiency, the ovaries of the RNAi group (N=3) were dissected at the end of the experiment. The interference efficiency of Mro-IR silencing was detected by quantitative real-time PCR (qPCR). SYBR Green RT PCR assay was carried out in a CFX384 quantitative PCR Detection System (Bio-Rad, US) for qPCR analysis as described (14). Mr-GAPDH (Genbank accession no. MH219928.1) was used as an internal reference to adjust the number of cDNA templates. The primers of Mr-GAPDH were designed to generate a 235 bp fragment of Mr-GAPDH and Mro-IR primers were designed to generate a 140 bp fragment of Mro-IR (**Supplementary Table 1**). A total volume of 20 ul mixture (10 ul of 2x SYBR Master Mix (Applied Biosystems, US), 1 ul of cDNA mix, 0.5ul of each primer (10 uM), and 8 ul of sterile distilled H_2_O was used for qPCR analysis according to the manufacturer’s protocol. And the program of qPCR was 95 ℃ for 1 min, followed by 40 cycles of 95 ℃ for 15 s and 63 ℃ for 25 s. Three replicates for each sample were performed. The relative expression level was calculated using the 2^-ΔΔCt^ method. The data obtained from qPCR analysis were analyzed for statistical significance using Graph-Pad Instat (GraphPad Software Inc.).

### 2.3 The histology of the ovary in Mro-IR knockdown

The effects of Mro-IR silence on the histomorphology and histology of proliferative ovary and premature ovary were explored, respectively. The injected prawns (N=3) of each group were placed in an ice bath for 2-3 min until lightly anesthetized. The ovary tissues were dissected for tissue section and hematoxylin-eosin (HE) staining. Each sample was fixed with 4% paraformaldehyde, dehydrated in gradient-increasing concentrations of ethanol, transparented in xylene, embedded in paraffin (Leica, HistoCore Arcadia), and sliced by paraffin slicer (Leica, HistoCore BIOCUT), respectively. The tissue section was cut at 5 μm thickness, stained with an HE staining kit (BBI), reviewed, and photographed under an optical microscope (Ningbo Yongxin Optics Co., Ltd., NOVEL, N-117M).

### 2.4 Comparative transcriptomic analysis of Mro-IR knockdown

To ascertain the effects of Mro-IR knockdown on the expression levels of related genes or factors in ovarian development and female reproduction, comparative transcriptomic profiling of female *M.rosenbergii* in response to Mro-IR silencing was enriched in this study.

The response of various related gene transcriptions and the effect of RNAi-mediated gene knockdown of Mro-IR on the expression of gonad-or reproductive-related genes were investigated by comparative transcriptomic analysis to evaluate the potential role of Mro-IR in the regulation of gonad development in *M. rosenbergii*. The ovary was dissected from gene-silencing females and the samples of each group were mixed (N=3) to provide sufficient RNA for the transcriptomic sequencing. Total RNA was extracted by using the column Trizol total RNA isolation kit (Order no. B511311, Sangon, Sangon Biotech (Shanghai) Co., Ltd., No. 698, Xiangmin Road, Songjiang District, Shanghai, China) following the manufacturer’s protocol. The OD260/280 should range from 1.8 to 2.0, to ensure the purity of the RNA sample.

The transcriptome was sequenced using the Illumina NovaSeq6000. The raw reads were cleaned by removing adaptor sequences, empty reads, and low-quality sequences. The clean reads were assembled into non-redundant transcripts, which have been developed specifically for the *de novo* assembly of the transcriptome using short reads. The resulting unigene sequences were then annotated using homology search (BLASTX) with an E-value cut-off of 10^-5^ against an NCBI non-redundant (Nr) database, Swissport, Cluster of Orthologous Groups database (COG), and Kyoto Encyclopedia of Genes and Genome (KEGG) database. The coding sequence and the direction of the annotated unigenes were determined based on the BLAST results from the four above-mentioned databases. For the differential expression analysis, the transcript expression level of the unigenes was measured using the FPKM method (Fragments Per kb per Million fragments). Genes were considered differentially expressed in the given library when the p-value was less than 0.05 and a greater than four-fold change (with the absolute value of log2foldchange more than 2).

Furthermore, the effect of RNAi-mediated gene knockdown of Mro-IR on the expressions of gonad-or reproduction-related genes was also investigated. The differentially expressed candidate genes were considered when the p-value was less than 0.05 and a greater than two-fold change (with the absolute value of log2foldchange more than 1) in comparative transcriptomic analysis. The putative cascade regulation axis or networks of these differentially expressed transcripts involved in crucial signal pathways of gonad development and reproduction were briefly illustrated.

### 2.5 Quantitative real-time PCR analysis

To validate the accuracy of gene expression data obtained by RNA-seq, several DEGs were selected to be verified by qPCR using the same samples for RNA-seq. The selected DEGs consisted of two up-regulated unigenes (male reproductive-related LIM protein (MRLIM) and sex-lethal (Sxl)) and eight down-regulated unigenes (vitellogenin receptor (VgR), low-density lipoprotein receptor (LDLR), lipid storage droplet protein (LSDP), insulin-like growth factor receptor (IGFR), JHE-like carboxylesterase (JHEC), heat shock protein 90 (HSP90), tyrosine kinase receptor (TKR) and Mro-IR) from the transcriptomic library of the proliferative ovary. Meanwhile, three up-regulated unigenes (forkhead box L2 (Foxl2), Sxl, apolipoprotein D (ApD)) and three down-regulated unigenes (zinc finger protein (ZFP), male reproductive-related protein (MRR) and Mro-IR) from the transcriptomic library of the premature ovary were selected. PCR Primers were designed and listed in **Supplementary Table 1**. Then Mr-GAPDH was used as a housekeeping gene to normalize the mRNA levels of DEGs. For qPCR analysis, total RNA from transcriptome sequencing samples was reverse-transcribed using SuperScript™ III First-Strand Synthesis SuperMix for qRT-PCR (Invitrogen). The SYBR Green RT PCR assay was carried out as above described. The qPCR was performed in triplicate. To confirm that only one PCR product was amplified and detected, a dissociation curve analysis of amplification products was performed at the end of each PCR reaction. The relative expression level was calculated using the 2^−ΔΔCt^ method. The data obtained from qPCR analysis and RNA-seq were calculated by POWER function and analyzed using Graph-Pad Instat (GraphPad Software Inc.).

## 3 Results

### 3.1 *In vivo* knockdown of Mro-IR by RNAi in female prawn *M.rosenbergii*

**Figure 1:**
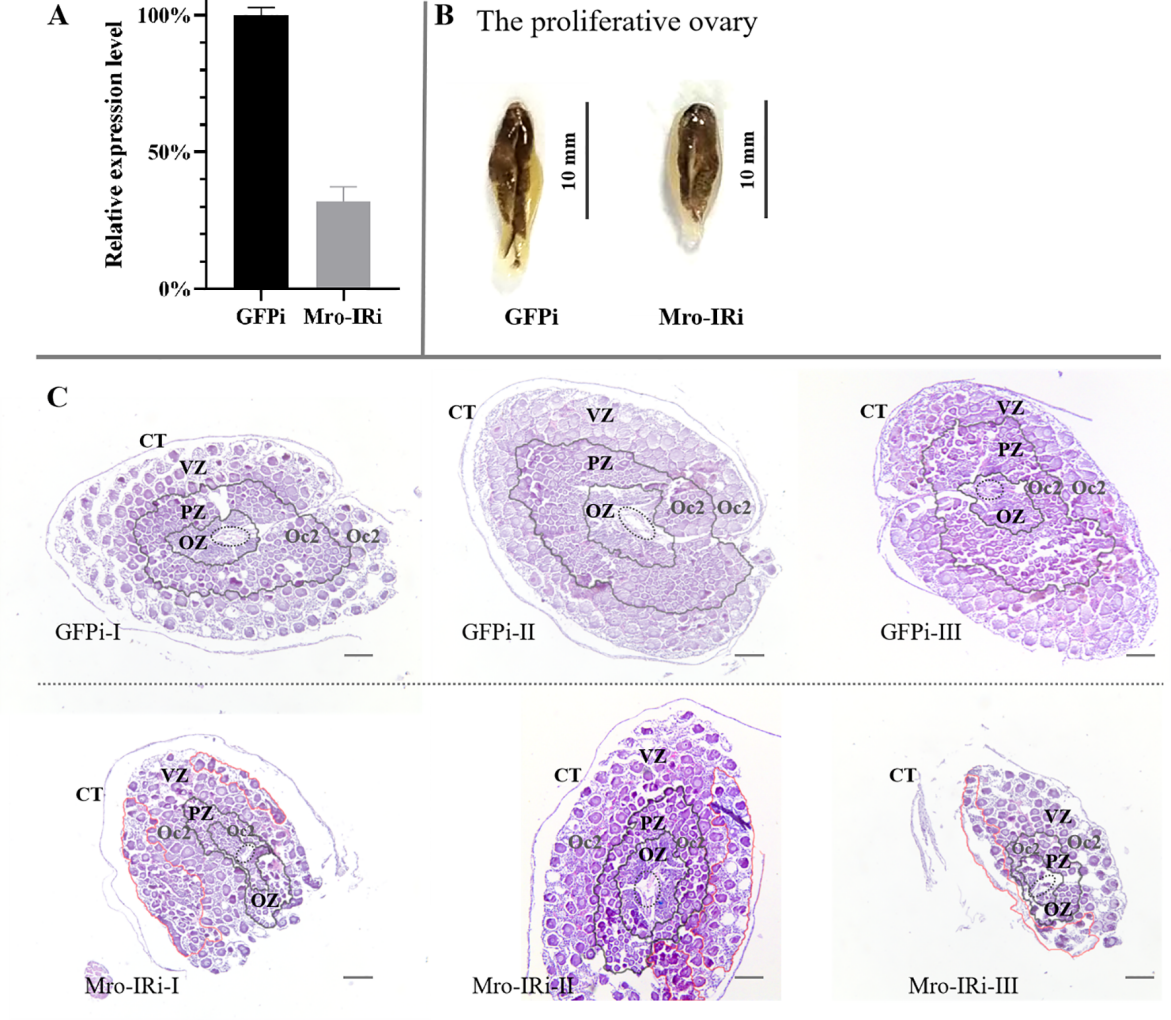
*In vivo* knockdown of Mro-IR by RNAi in the stage of the proliferative ovary in females. **(A)** Detection of the relative Mro-IR mRNA level in RNAi induced and the control group. The histogram of the relative Mro-IR mRNA expression level in RNAi-induced and control groups. The expression of Mro-IR was reduced to 35% in the female reproductive system at the end of the experiment. **(B)** The appearance of the proliferative ovary in RNAi-mediated females *M. rosenbergii*. The dorsal surface of the proliferative ovary was gray. **(C)** The histology of the proliferative ovary of Mro-IR-silenced prawns. The ovarian follicle is clearly outlined by the connective tissue trabeculae. The elliptical regions with the dashed line indicated the central core (CC). As shown in the circle area with the red line, some Oc2 presented abnormal cellular morphology with intense basophilic cytoplasm by HE staining in PZ in the knockdown of the Mro-IR group. OZ, oogenic zone; PZ, previtellogenic zone; VZ, vitellogenin zone; Oc2, early vitellogenic oocytes. Bar: 300 um.

*In vivo* knockdown of Mro-IR gene was carried out in adult female prawns to evaluate the potential function of Mro-IR in gonad development and reproduction, especially in the proliferative ovary (stage II) and premature ovary (stage III), respectively. On one hand, the mRNA expression level of Mro-IR was confirmed to dramatically decreased to less than 35% compared with control samples during the proliferative ovary stage in the RNAi experiment (**Figure 1A**).The ovarian appearance of Mro-IR-silenced individuals morphologically resembled that of control females. As shown in **Figure 1B**, the dorsal surface of the proliferative ovary was gray in color, some small and yellow doughnut-shaped masses appeared in the GFP RNAi group. Histologically, the ovary was enwrapped by a connective tissue membrane (also known as ovarian membrane). In the control group (GFP RNAi group), the central core (CC) containing layers of oogonia and the peripheral zones (primarily contained with ring and ring evenly distributed oogenic zone (OZ), previtellogenic zone (PZ) and vitellogenic zone (VZ)) of proliferative ovary were illustrated in **Figure 1C**. Various reproductive cells, such as oogonia cells (Oo), previtellogenic oocytes (Oc1), and early vitellogenic oocytes (Oc2) were arrayed in order in respective zones. Many Oc2 regularly arrayed in the out peripheral zone of the proliferative ovary and formed distinct rings of PZ and VZ in the control group (**Figure 1C** GFPi). Differently, a smaller number of Oc2 were irregularly found both in relatively narrowed PZ and enlarged VZ in the Mro-IR-silenced ovary (**Figure 1C** Mro-IRi), which indicated the proliferation of Oc2 was significantly delayed or inhibited in Mro-IR silence.

**Figure 2:**
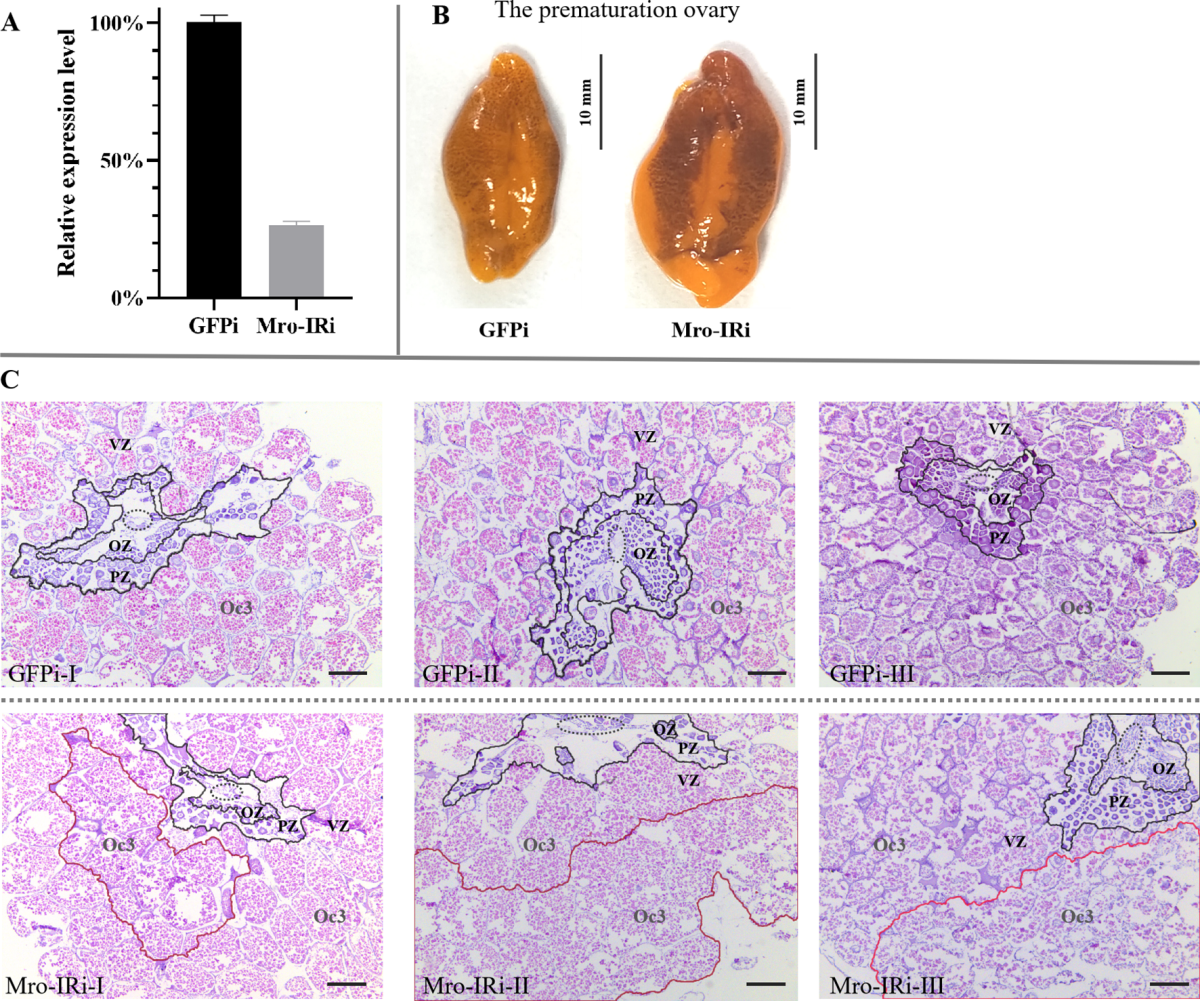
*In vivo* knockdown of Mro-IR by RNAi in the stage of the prematuration ovary in females. **(A)** Detection of the relative Mro-IR mRNA level in RNAi induced and the control group. The histogram of the relative Mro-IR mRNA expression level in RNAi-induced and control groups. The expression of Mro-IR was reduced to 25% in the female ovary at the end of the experiment. **(B)** The appearance of the prematuration ovary in RNAi-mediated female prawns. The significantly enlarged ovary developed a characteristic orange-green color. **(C)** The histology of the premature ovary of Mro-IR-silenced prawns. The elliptical regions with the dashed line indicated the central core. As shown in the circle area with the red line, the abnormal Oc3 oocytes showed fragmented borders and presented the spilled eosinophilic cytoplasm in the knockdown of the Mro-IR group. OZ, oogenic zone; PZ, previtellogenic zone; VZ, vitellogenin zone; Oc3, late vitellogenic oocytes; Bar: 300 um.

On the other hand, the transcription level of Mro-IR was significantly reduced to 25% compared with that of the GFP RNAi group during the premature ovary stage (**Figure 2A**). As shown in **Figure 2B**, the premature ovary increased significantly in size and developed a characteristic orange-green color. Histologically, the oocytes radially and continuously migrated towards the periphery of the ovary. The premature ovary was subdivided into cone-shaped ovarian pouches with a CC, thinned OZ and PV, and an enlarged VZ with the primary late vitellogenic oocytes (Oc3) in the peripheral zone in the GFP RNAi group (**Figure 2C** GFPi). Significantly, there were a smaller number of Og or oocytes (Oc1 and Oc2) in the CC, OZ and PV, and a high proportion of abnormal Oc3 with indistinct boundary in the VZ in Mro-IR-silenced female prawn (**Figure 2C** Mro-IRi).

### 3.2 The comparative transcriptomic analysis of Mro-IR knockdown

#### 3.2.1 The comparative transcriptomic analysis of Mro-IR knockdown in the proliferative ovary

**Figure 3:**
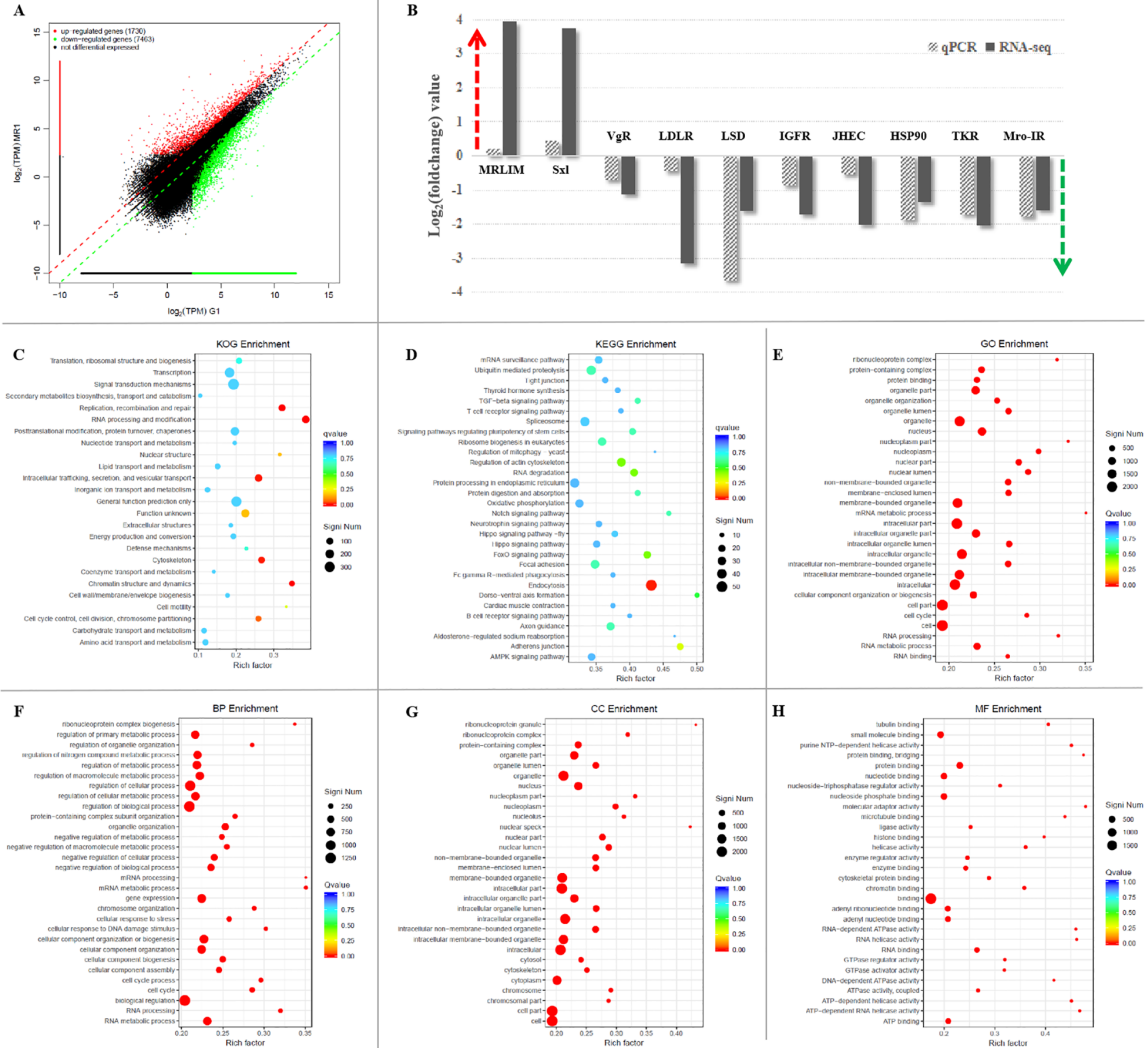
The comparative transcriptomic analysis of proliferative ovary in Mro-IR knockdown. **(A)** The significant differentiation of upregulated and downregulated genes in the Mro-IR RNAi group vs GFP RNAi group. The 1730 red dots indicated the upregulated unigenes and the 7463 green dots indicated the downregulated unigenes, respectively. **(B)** Verification of the expression patterns both in real-time quantitative reverse transcription PCR and RNA-seq. The ten detected genes showed similar upregulated or downregulated expression patterns, respectively. The results indicated the reliability and accuracy of our transcriptome analysis. MRLIM, male reproductive-related LIM protein; Sxl, sex-lethal; VgR, vitellogenin receptor; LDLR, low-density lipoprotein receptor; LSD, lipid storage droplet protein; IGFR, insulin-like growth factor receptor; JHEC, JHE-like carboxylesterase; HSP90, heat shock protein 90; TKR, tyrosine kinase receptor. **(C)** KOG enrichment of DEGs in Mro-IR RNAi group vs GFP RNAi group. 1. replication, recombination, and repair, 2. RNA processing and modification, 3. intracellular trafficking, and vesicular transport, 4. cytoskeleton, and 5. chromatin structure and dynamics of enriched DEGs were found to be dominant. **(D)** KEGG enrichment of DEGs in Mro-IR RNAi group vs GFP RNAi group. The endocytosis of enriched DEGs was found to be dominant. **(E)** Gene Ontology (GO) enrichment of DEGs in Mro-IR RNAi group vs GFP RNAi group. **(F)** Biological process (BP) enrichment of DEGs. **(G)** Cellular component (CC) enrichment of DEGs. **(H)** Molecular function (MF) enrichment of DEGs.

The transcriptomic libraries obtained about 33.73 million clean reads and 4.72G clean bases in the Mro-IR RNAi group, and 33.97 million clean reads and 4.78G clean bases in the GFP RNAi group in the proliferative ovary. The values of Q20 and Q30 were more than 98.5% and 95.0% in both groups, respectively. Firstly, differential gene expression (DGE) analysis between Mro-IR silencing and the control group generated 9193 genes of which, 1730 unigenes were up-regulated and 7463 unigenes were down-regulated (**Figure 3A**). Furthermore, ten DEGs (two up-regulated unigenes and eight down-regulated unigenes) were selected for qRT-PCR validation. As a result, all the detected genes showed similar trends of expression patterns with those of RNA-seq (**Figure 3B**), which indicated the reliability and accuracy of our transcriptome analysis.

Secondly, the top eight representative groups of pathways with a higher percentage of differentially regulated genes were clustered into endocytosis, PI3K-Akt signaling pathway, ubiquitin-mediated proteolysis, spliceosome, protein processing in the endoplasmic reticulum, RNA transport, regulation of actin cytoskeleton, and focal adhesion. Meanwhile, the highest numbers of up-and down-regulated unigenes were focused on protein kinase and the higher amounts of differentially regulated genes were enriched on reverse transcriptase, RNA recognition motif, zinc-finger double domain, trypsin, Ankyrin repeats, BTB/POZ domain, and glycosyl hydrolases family, respectively.

Then, the transcripts (with significant differential expression) were subjected to DEG-enriched pathway analysis through the KOG database, KEGG database, and gene ontology (GO) database, respectively. As shown in the KOG enrichment in **Figure 3C**, differential genes were significantly enriched in five classifications: 1. replication, recombination, and repair, 2. RNA processing and modification, 3. intracellular trafficking, and vesicular transport, 4. cytoskeleton, and 5. chromatin structure and dynamics, respectively. Moreover, endocytosis was the exclusive pathway with significantly enriched differential unigenes by KEGG database analysis (**Figure 3D**). In addition, there were many significant differentials expressed transcripts classified into various pathways in GO enrichment analysis (**Figure 3E**). Furthermore, the top thirty enriched pathways involved in biological process (BP) enrichment (**Figure 3F**), cellular component (CC) enrichment (**Figure 3G**), and molecular function (MF) enrichment (**Figure 3H**) were shown, respectively.

#### 3.2.2 The comparative transcriptomic analysis of Mro-IR knockdown in the premature ovary

**Figure 4:**
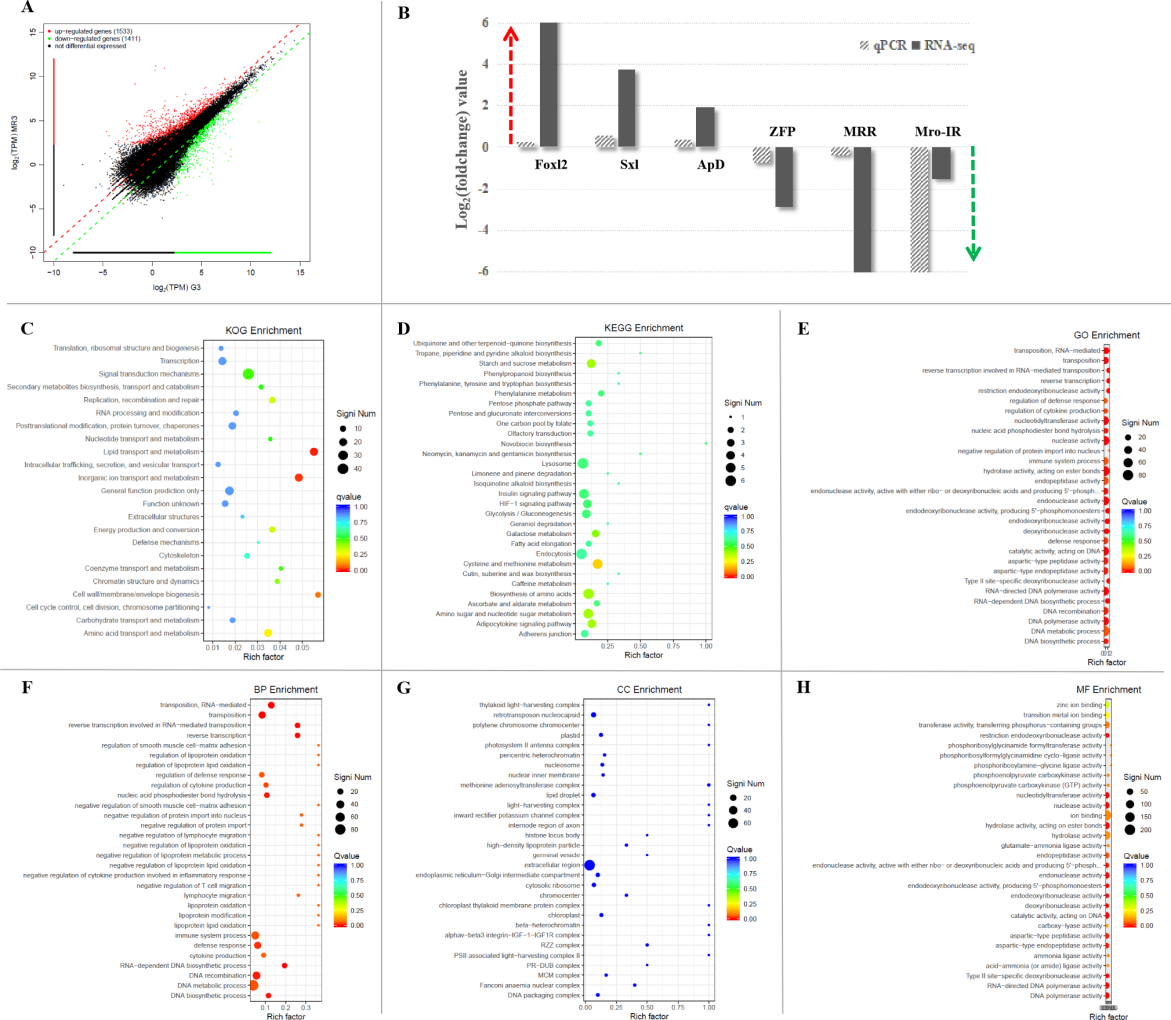
The comparative transcriptomic analysis of premature ovary in Mro-IR knockdown. **(A)** The significant differentiation of upregulated and downregulated genes in the Mro-IR RNAi group vs GFP RNAi group. The 1533 red dots indicated the upregulated unigenes and the 1411 green dots indicated the downregulated unigenes, respectively. **(B)** Verification of the expression patterns both in real-time quantitative reverse transcription PCR and RNA-seq. The six detected genes showed similar upregulated or downregulated expression patterns, respectively. The results indicated the reliability and accuracy of the transcriptome analysis. Foxl2, forkhead box L2; Sxl, sex-lethal; ApD, apolipoprotein D; ZFP, zinc finger protein; MRR, male reproductive-related protein. **(C)** KOG enrichment of DEGs in Mro-IR RNAi group vs GFP RNAi group. 1. lipid transport and metabolism, 2. inorganic ion transport and metabolism, and 3. cell wall/membrane/envelope biogenesis were found to be dominant. **(D)** KEGG enrichment of DEGs in Mro-IR RNAi group vs GFP RNAi group. **(E)** Gene Ontology (GO) enrichment of DEGs in Mro-IR RNAi group vs GFP RNAi group. **(F)** Biological process (BP) enrichment of DEGs. **(G)** Cellular component (CC) enrichment of DEGs. **(H)** Molecular function (MF) enrichment of DEGs.

The transcriptomic libraries obtained about 29.24 million clean reads and 3.92G clean bases in the Mro-IR RNAi group and 30.89 million clean reads and 4.20G clean bases in the GFP RNAi group in the premature ovary. The values of Q20 and Q30 were also more than 98.5% and 95.0% in both groups, respectively. Firstly, DGE analysis between Mro-IR silencing and the control group generated 2944 genes of which, 1533 unigenes were up-regulated and 1411 unigenes were down-regulated (**Figure 4A**). In the present study, six DEGs (three up-regulated unigenes, Foxl2, Sxl, and ApD, and three down-regulated unigenes, ZFP, MRR, and Mro-IR) were selected for qRT-PCR validation. As a result, all the detected genes showed similar trends of expression patterns with those of RNA-seq (**Figure 4B**), which indicated the reliability and accuracy of our transcriptome analysis.

Secondly, the top eight representative groups of pathways with a higher percentage of differentially regulated genes were clustered into the biosynthesis of amino acids, lysosome, endocytosis, cysteine and methionine metabolism, amino sugar and nucleotide sugar metabolism, insulin signaling pathway, PI3K-Akt signaling pathway, and purine metabolism. Meanwhile, the highest numbers of up-and down-regulated unigenes were focused on reverse transcriptase and the higher amounts of differentially regulated genes were enriched on ligated ion channel, superfamily endonuclease, cytochrome P450, integrase core, and trypsin, respectively.

Then, the significant differential expression transcripts were synchronously subjected to DEG-enriched pathway analysis through the KOG database, KEGG database, and GO database, respectively. As shown in the KOG enrichment in **Figure 4C**, differential genes were significantly enriched in three classifications: 1. lipid transport and metabolism, 2. inorganic ion transport and metabolism, and 3. cell wall/membrane/envelope biogenesis, respectively. Additionally, there was less information on DEG enrichments obtained from the KEGG (**Figure 4D**).

To gain insights into the biological processes, we have subjected significant differentially expressed transcripts to gene ontology (GO) enrichment analysis (**Figure 4E**). The GO terms were significant (p-value<0.05) in biological process (**Figure 4F**), cellular component (**Figure 4G**), and molecular function (**Figure 4H**). Within three main categories of GO classification, the top thirty enriched pathways involved in biological process (BP) enrichment (**Figure 4F**) were found to be dominant.

By comparison, the total data of the comparative transcription profiling of proliferative ovaries was much more abundant than that of the premature ovary. The number of enriched significantly differentially expressed unigenes in the proliferative ovary was approximately threefold than that in the premature ovary. Coincidently, both endocytosis and PI3K-Akt signaling pathways had been identified as the representative groups of pathways with a higher percentage of differentially regulated genes.

### 3.3 The response of ovarian development and reproduction-related candidate genes to Mro-IR silencing

**Figure 5:**
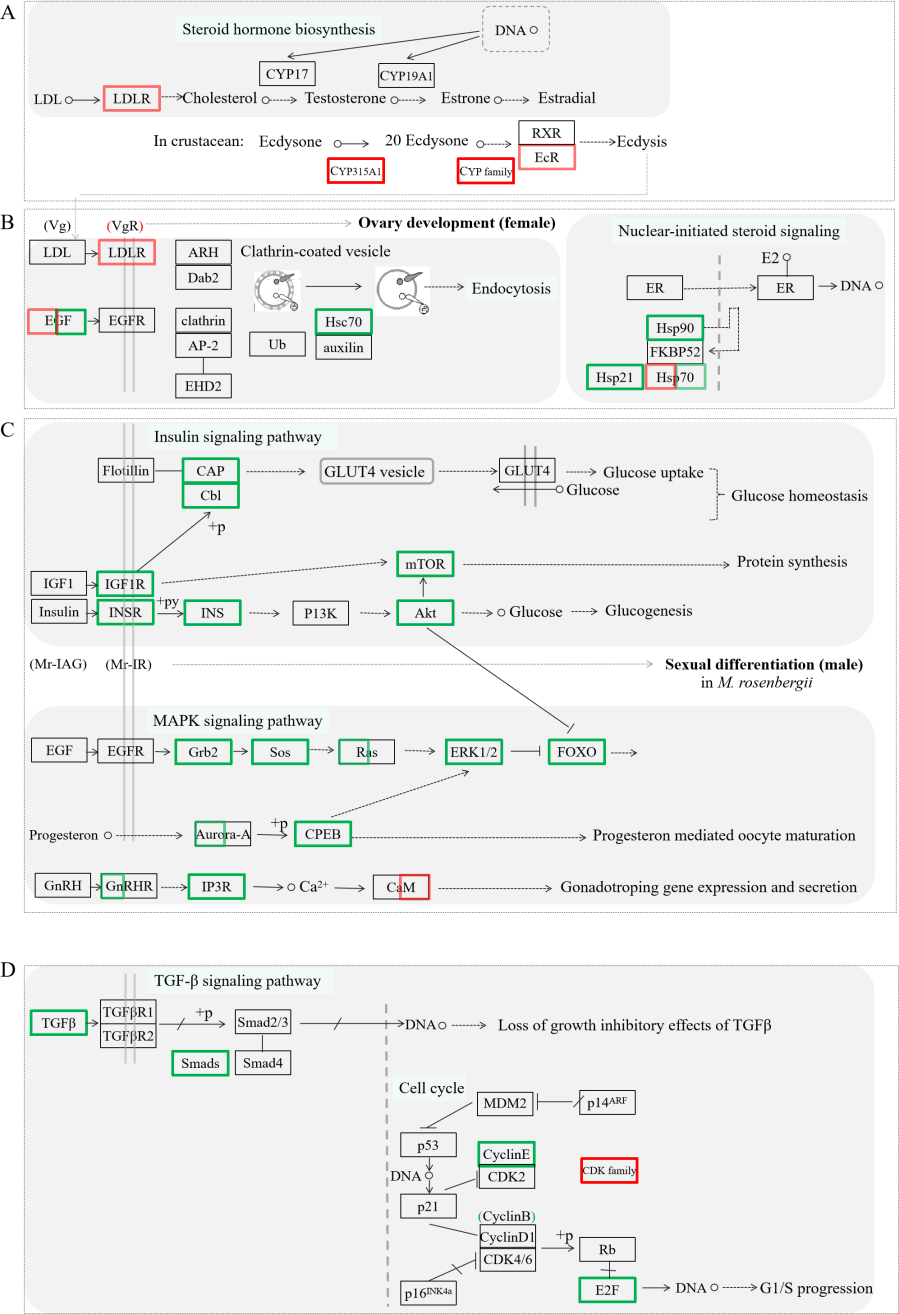
The significant differentially expressed unigenes enriched KEGG pathway analysis by the comparative transcriptomic analysis of Mro-IR knockdown. Several kinds of categories with a higher percentage of upregulated and downregulated transcripts were primarily focused on the pathways of steroid hormone biosynthesis involved ecdysis **(A)**, endocytosis, and nuclear-initiated steroid signaling participated in ovarian development **(B)**, some signaling pathways including insulin and IGF signaling pathway, MAPK signaling pathway and GnRH signaling pathway, related to glycometabolism and proteometabolism **(C)**, and TGF-b signaling pathway and cell cycle **(D)**, respectively. The primary upregulated transcripts (red color frame) and the downregulated transcripts (green color frame) were briefly profiled in the various signaling pathways.

The cell division and proliferation of gamete and substance accumulation are crucial events during ovarian development. To elucidate the potential regulatory mechanism of Mro-IR gene in ovarian development and female reproduction, the putative intuitive cascade regulation axis or networks of the vital differentially expressed transcripts involved in crucial signal pathways of gonad differentiation or cell proliferation, such as steroid hormone biosynthesis (**Figure 5A**), ovarian development (**Figure 5B**), nuclear-initiated signaling (**Figure 5B**), insulin signaling pathway (**Figure 5C**), MAPK signaling pathway (**Figure 5C**), TGF-β signaling pathway (**Figure 5D**), and cell cycle (**Figure 5D**), were briefly illustrated. To gain insights into the biological processes being operative during ovarian development and reproduction in *M. rosenbergii* including other differentially expressed genes, we have subjected transcripts (with significant differentially expressed) to DEG-enriched KEGG pathway analysis. Seven kinds of categories with a higher percentage of up-and down-regulated unigenes were primarily focused on the pathways of ovarian steroidogenesis, estrogen signaling pathway, insulin signaling pathway, FOXO signaling pathway, endocytosis, oocyte meiosis, and GnRH signaling pathway, respectively. The influences or changes of these up-regulated and down-regulated transcripts were presented in **Supplementary Tables 2**, **3**.

In conclusion, the comparison of gene transcripts between Mro-IR knockdown and control females was greatly helpful in providing evidence of the possible related candidate genes involving ovarian development and other biological processing in the present study.

## 4 Discussion

Mro-IR is an ovary-uniquely expressed insulin receptor and gradually increased with the ovarian development of the female prawn *M. rosenbergii* (14). In the present study, the possible molecular mechanism of Mro-IR involved in the ovarian development and female reproduction of *M. rosenbergii* was explored. Firstly, the effects of Mro-IR silencing in cell proliferation and vitellogenesis during proliferative ovary and premature ovary were explored by histological analysis, respectively. The results showed that the Mro-IR knockdown significantly inhibited the cell proliferation of germ cells, caused abnormal cellular morphology of Oc2, and disturbed the yolk accumulation process in Oc3. It was suggested that the Mro-IR played significantly promoting roles in oocyte development (such as Oc2 and Oc3) during the ovarian development in female prawn *M.rosenbergii.*

Moreover, the comparative transcriptomic analysis provided more detailed data to help clarify the molecular mechanism of Mro-IR participated in ovarian development in prawns.

### 4.1 Cell division cycle

In the present study, some cell cycle-related genes, such as Aurora, cyclin-dependent kinases 2 (CDK2), Rac8, and transforming growth factor-β (TGF-β) unigenes, showed a significant down-regulation trend in Mro-IR knockdown, which suggested that Mro-IR have a promoting or maintaining effect in mitosis/meiosis. Cyclin-dependent kinases (CDK) family genes are considered to be key factors in the cell cycle. In *M. rosenbergii*, Cdk2 transcription has a high expression level in meiosis spermatocytes and oocytes, which is considered to be functionally conservative (18).

In addition, it was found that the Mro-IR gene was expressed through the whole embryonic development of *M. rosenbergii* with higher transcript levels in the nauplius period and gastrula period, but lower expression levels in fertilized egg period and zoea period (data not shown). That was to say Mro-IR was involved in mitosis during embryonic development and probably had to promote roles in the processes of cell differentiation and organogenesis.

### 4.2 Steroid signaling for ecdysis

#### 4.2.1 Cytochrome P450 family

In ecdysozoans, development and reproduction are regulated by ecdysteroids or molting hormones, which are synthesized from dietary cholesterol by steroidogenic enzymes of the cytochrome P450 (CYP) family. In the present study, some members of CYP super-family genes, CYP315a1, CYP2, and CYP2G1 homologs from in the premature ovary and CYP302a1 homolog from the proliferative ovary were enriched as significantly downregulated in the Mro-IR-silencing female. The knockdown of ecdysteroidogenic enzyme genes (CYP306A2, CYP314A1, CYP315A1, and CYP302A1) in newly molted females of the rice planthopper *Nilaparvata lugens*, caused the failure of egg production: less vitellogenic and mature eggs in ovaries, fewer laid eggs and embryonic development deficiency of laid eggs (14, 19).

#### 4.2.2 Molting-related factors

Many genes or factors cooperatively play vital roles in the regulation of growth and reproductive development, including morphogenesis, metamorphosis, reproduction, molting, and the response to stress in crustaceans (20–22). In the present study, some molting-related factors, such as the upregulated juvenile hormone binding protein (JHBP) and lipocalin homologs, the downregulated homologs, ecdysone receptor (EcR), juvenile hormone epoxide hydrolase (JHEH), some CHH family unigenes, chitin binding preritropin (CBP), chitin synthesis, calcium-activated chloride channel (CLCA) and methyltransferase (MTS), and several significant differentiated expressed ion-channels were also enriched in the Mro-IR knockdown. It suggested that Mro-IR probably played an important role in corporately participating in ovarian development and molting.

#### 4.2.3 Heat shock protein

Heat shock protein (Hsp) contributes to the interaction with steroid hormone receptors, temperature, estrogen signaling, etc., and several Hsps are critical for successful embryogenesis and reproduction (23, 24). In the present study, downregulated Hsp90 homologs and significant differentially expressed Hsp70 homologs were enriched in the ovarian comparative transcriptomic analysis of Mro-IR silence. Both of them are important components of the Hsp90-FKBP52-Hsp70 complex which involve in nuclear-initiated steroid signaling (23). Thus, Mro-IR silence displayed significant downregulated effects on the expressions of Hsp90 and Hsp21, and influence on the transcription of Hsp70s, which suggested Mro-IR probably plays a complex regulatory role in the Hsp family.

### 4.3 Endocytosis for ovarian development

The process of yolk synthesis or vitellogenesis is the key event in oocyte development (15, 25). Vitellogenesis in *M. rosenbergii* begins with the synthesis of vitellogenin (Vg) in the hepatopancreas, then it is released into the hemolymph and cleaved into vitellin (Vn), which is taken up through receptor-mediated endocytosis (RME) and incorporated into the yolk granules in the late oocytes, i.e., Oc3 and Oc4 (15, 26, 27). It is known that either Vg or VgR participates in the promoting of ovarian development and maturation. For instance, in *P. vannamei*, Lv-VgR gene knockdown resulted in the arrest of oocytes in the previtellogenesis oocyte stage, which almost contained no vitelloprotein (28). In the present study, the crucial elements of vitellogenesis, such as downregulated vitellogenin unigene and Hsc70, some significantly upregulated low-density lipoprotein receptor unigene (LDLR, vitellogenin receptor homolog) and significant differentially expressed epidermal growth factor (EGF) homologs were enriched in the comparative transcriptomic library in Mro-IR silencing, which suggested that the Mro-IR had a significant promoting or maintain the effect on the female ovarian development.

Moreover, some proteinases were also generated as significantly differential expression transcripts from the ovary of Mro-IR knockdown. For instance, cathepsin is a proteinase hydrolyzing Vg into Vn. In the present study, several cathepsin homologs (also annotated as peptidase homologs) were enriched as downregulated unigenes and many proteins containing the domains of ankyrin or ubiquitins were identified in the ovarian in the Mro-IR knockdown. In *M. rosenbergii*, the Mrfem-1 presented eight ankyrin repeats and was exclusively expressed in the ovary of the giant freshwater prawn (29). It was located in the cytoplasm of the previtellogenic stage and scattered in the cytoplasm and follicular cells at the vitellogenic stage (29). For another instance, Mnfem-1 is an ovary-specific gene from the oriental river prawn, *Macrobrachium nipponense* (30). The Mnfem-1 protein can potentially interact with cathepsin L and proteins containing the domains of insulinase, ankyrin, or ubiquitin (30). Meanwhile, several trypsin homologs were also enriched as significantly downregulated transcripts, which suggested that the Mro-IR regulates the Vg degradation in ovarian development.

### 4.4 Protein synthesis and degradation pathways

Insulin and insulin-like growth factor (IGF) signaling systems are ancient and involved in growth, development, cell differentiation, and metabolism (1, 31). These signaling systems comprise respective ligands, receptors, binding proteins, and hydrolases (31). In the present study, some potential factors, such as insulin growth factor1 receptor (IGF1R), insulin synthesis receptor (INSR), and insulin synthesis (INS), were enriched as downregulated homologs in Mro-IR knockdown. It was indicated that Mro-IR probably had a promotion effect on the insulin or insulin-like signaling pathway, which may play a regulatory role in female gonad development.

Degradation operates via one of the two general protein degradation pathways: the ubiquitin-proteasome system (UPS), or autophagy. In the present study, the ubiquitin carboxyl-terminal hydrolase, ubiquitin-conjugating enzyme, and cullins homologs were significantly decreased in Mro-IR, which strongly suggested a tight link between the Mro-IR and the UPS. Meanwhile, several downregulated unigenes, Ras, mitogen-activated protein kinase (MAPK), inositol 1,4,5-triphosphate receptor (IP3R), Caspases 8 (Casp8), α-tubulin and cathepsin homologs, were identified in the pathway of apoptosis in the proliferative ovary in the Mro-IR knockdown. Moreover, the downregulated nuclear factor-kappa-B inhibitor alpha (IkBα) unigene in the nuclear factor-kappa-B (NF-kB) signaling pathway was enriched. That meant the Mro-IR played an important role in maintaining the autophagy process in ovarian development. In brief, the Mro-IR has a positive role in ovarian development and would participate in ovarian maturation through a complex network involving vitellogenesis and protein degradation pathways.

### 4.5 Sexual regulation-related genes

In crustaceans, the endocrine axis of the eyestalk-AG-gonad (testis/ovary) is considered to be a key pathway in determining sexual development (3, 32). In the present study, a significantly decreased CHH family gene and a down-regulated neuroparsin gene were found in Mro-IR knockdown. These results demonstrated that Mro-IR was associated with neuropeptides and had distinct effects on the molt and gonad development in crustaceans.

The insulin-like signaling pathway is predominantly thought to play a regulatory role in sexual differentiation among crustaceans (3). In *M. rosenbergii*, Mr-IR is the receptor of Mr-IAG and participated in the male sexual differentiation whereas the silencing of Mr-IR caused a full and functional sex reversal species (12, 13). Moreover, the mRNA of Mr-IAG binding protein (Mr-IAGBP) was detected in a wide array of tissues with the highest expression found in the androgenic gland in *M. rosenbergii* (33). The injection of Mr-IAGBP dsRNA significantly reduced the transcription of Mr-IAG, while the amount of Mr-IAGBP mRNA and the translation of IAGBP protein was significantly reduced by the injection of Mr-IAG dsRNA, which revealed that IAGBP is involved in IAG signaling (33). In the present study, a significantly reduced expression of insulin-like peptide (ILP), a down-regulated IAGBP, and another down-regulated insulin-like receptor gene (different from Mro-IR and Mr-IR) were detected in the ovary after the interference of the Mro-IR gene. These data supported the hypothesis that (IAG/ILP and IAGBP)-IAG receptor/IR signaling schemes exist in *M. rosenbergii*. This novel evidence showed that the insulin-like signaling pathway participated in female sexual differentiation and plays an important role in gonad development and reproduction.

Forkhead box L2 (Foxl2) is a member of the Fox gene family and a conserved transcription factor that is defined by a unique DNA-binding domain (34). *Foxl2* is a special marker of ovarian differentiation and plays a critical role in ovarian differentiation and maintenance (35, 36). *Foxl2* displayed upregulated expression in the testis compared with that in the ovary in *M. rosenbergii* (37). In the present study, some downregulated Foxl2 homologs were enriched in the ovary of Mro-IR knockdown, which indicated the potential corresponding role of Mro-IR in promoting the activity of Foxl2 homologs.

MroDmrt11E is a member of the doublesex and mab-3 related transcription factor (Dmrt) family genes and participated in the sexual differentiation in *M. rosenbergii* (17). It is noteworthy that the knockdown of MroDmrt11E dramatically decreased the transcription of Mro-IR (17). Moreover, MroDmrt11E have depression effects on the transcripts of Foxl2, Nr5a2, and CYP315a1 homologs in MroDmrt11E knockdown in male reproductive (17). Thus, Mro-IR probably was an important factor in ovarian maturation and molt by regulating the expressions of Foxl2, CYP315a1, CHH family, and EcR homologs, whereas their transcripts were significantly inhibited in Mro-IR knockdown. This information provided novel clues to understand the potential mechanism of the eyestalk-AG-gonad (testis/ovary) endocrine axis involved in the coordinated regulation network among the molt, sex, and reproduction processes.

In conclusion, Mro-IR played a significant and positive effect on the proliferation of Oc2 and Oc3 oocytes, and promoted the yolk accumulation during ovarian development in female prawn *M. rosenbergii*. Meanwhile, the related candidate genes and putative intuitive cascade regulation axis or networks of Mro-IR involved in crucial signal pathways of gonad development and reproduction were explored and briefly illustrated. This finding provided a novel basis for elucidating the molecular mechanism of Mro-IR in oocyte proliferation in ovarian development in *M. rosenbergii*.

## Data Availability

The authors acknowledge that the data presented in this study must be deposited and made publicly available in an acceptable repository, prior to publication. Frontiers cannot accept an article that does not adhere to our open data policies.
