# Supplementary material for: Involvement of the ovarian-specific Mro-IR in oogonia differentiation and oocyte development in freshwater giant prawn Macrobrachium rosenbergii
Source: Front Endocrinol (Lausanne). 2025 Apr 3;16:1516849. doi: 10.3389/fendo.2025.1516849 (PMC12004233; doi:10.3389/fendo.2025.1516849)
Supplement: Supplementary file 1 [file Table1.docx]

Supplementary table 1 Nucleotide sequences and information of primers used in PCRs.

| **Gene Description** | **Gene ID** | **Primer description** | **Primer sequence (5’-3’)** | **Primer length (bp)** | **Fragment length (bp)** |
| --- | --- | --- | --- | --- | --- |
| GAPDH | MH219928.1 | Mr-GAPDH-qF | GTGCCGCCCAGAACATCAT | 19 | 235 |
|  |  | Mr-GAPDH -qR | TCTTCGGTGTAGCCCAGGA | 19 |  |
| Mro-IR | OP966788.1 | Mro-IR-qF | GAGGGAGCGTCAGGATAC | 18 | 140 |
|  |  | Mro-IR-qR | GGCTGAGTTGCAACGATTGC | 20 |  |
| Lipid storage droplet protein | TRINITY_DN41228_c11_g2 | LSD-qF | TCGCCAGAGCAGTTCGT | 17 | 145 |
|  |  | LSD-qR | CGGTTGTCAGGTCTTCCAC | 19 |  |
| Low-density lipoprotein receptor | TRINITY_DN40799_c1_g1 | LDLR-qF | TGATTGCACGAGCGAACT | 18 | 135 |
|  |  | LDLR-qR | TCGCAACGCAACCACCT | 17 |  |
| Tyrosine kinase receptor | TRINITY_DN31498_c0_g1 | TKR-qF | GAAAAGCAGGGGCAGACC | 18 | 145 |
|  |  | TKR-qR | GCTCGTAGAGTTAGCGTGTAGTT | 23 |  |
| Sex-lethal | TRINITY_DN38558_c0_g3 | Sxl-qF | CAGATCCTACACCCTAGACCAAT | 23 | 96 |
|  |  | Sxl-qR | GGTAACCCAGTAACCTTGTCCTT | 23 |  |
| Apolipoprotein D | TRINITY_DN31871_c1_g6 | ApD-qF | GGAGCCTAAACTCAGCAATACG | 22 | 181 |
|  |  | ApD-qR | CTTAGCCTTCTTGGGGTCC | 19 |  |
| Forkhead box L2 | TRINITY_DN35254_c1_g3 | FoxL2-qF | ATCAGGCACAACCTCAGTC | 19 | 196 |
|  |  | FoxL2-qR | GGTAGGTCTCTGGGTAAGTGT | 21 |  |
| Male reproductive-related LIM protein | TRINITY_DN37886_c1_g1 | MRLIM-qF | CAAAGTGCGGCAAGTCC | 17 | 139 |
|  |  | MRLIM-qR | TAAAGGACGTTCTCGTG | 17 |  |
| JHE-like carboxylesterase | TRINITY_DN40120_c1_g4 | JHEC-qF | ATGTGTGCGTGCGTGAC | 17 | 121 |
|  |  | JHEC-qR | CAGCAATGGAGCAACCC | 17 |  |
| Vitellogenin receptor | TRINITY_DN40884_c2_g3 | VgR-qF | CAACGGGGATCTCGACTG | 18 | 60 |
|  |  | VgR-qR | CATCTTGAGGTCTCGCAGTG | 20 |  |
| Zinc finger protein | TRINITY_DN32195_c2_ | ZFP-qF | GCAGAAGAAGAGGACGACCAG | 21 | 97 |
|  |  | ZFP-qR | GGCGAGCAGGAATAGCAA | 18 |  |
| Male reproductive-related protein | TRINITY_DN27880_c0_g1 | MRR-qF | CACAAGCCTAAGCCTACCG | 19 | 225 |
|  |  | MRR-qF | TGACCCAACACTCTGCCTAAC | 21 |  |
| Insulin-like growth factor receptor | TRINITY_DN40945_c4_g1 | IGFR-qF | AGGACCCACGGAGTCATAGA | 20 | 139 |
|  |  | IGFR-qR | GGTTCGTGGTCGTGCTCAT | 19 |  |
| Heat shock protein 90 | TRINITY_DN38649_c1_g1 | HSP90-qF | TTCCTCCGCTACCACACC | 18 | 239 |
|  |  | HSP90-qR | AGCTGCTTGCCATCATACTCC | 21 |  |

F and R indicate the forward and reverse directions respectively.

Supplementary table 2 Significantly differential expression of molt, reproduction and sex-related candidate genes in the comparative transcriptomic analysis of proliferative ovary in Mro-IR knockdown

| **Gene Description** | **Gene ID** | **MeanTPM**  **(Mro-IRi)** | **MeanTPM**  **(GFPi)** | **log2Fold**  **Change** | **pValue** | **qValue** | **Result** | **GenBank No.** | **Species** |
| --- | --- | --- | --- | --- | --- | --- | --- | --- | --- |
| Calcium binding and coiled-coil domain | TRINITY_DN40165_c1_g3 | 28.64386 | 113.9898 | -1.99261 | 1.63×10^-50^ | 5.90×10^-49^ | down | - | *-* |
| Chitin binding Peritrophin-A domain | TRINITY_DN35411_c0_g1 | 1.746056 | 13.0949 | -2.90683 | 1.03×10^-8^ | 7.88×10^-8^ | down | CBY38644.1 | *Oikopleura dioica* |
| Chitin binding Peritrophin-A domain | TRINITY_DN40023_c0_g1 | 2.269765 | 11.10115 | -2.29009 | 2.03×10^-7^ | 1.37×10^-6^ | down | XP_013775794.1 | *Limulus polyphemus* |
| Carbohydrate | TRINITY_DN37276_c1_g1 | 21.80799 | 45.67853 | -1.06666 | 1.73×10^-61^ | 7.72×10^-60^ | down | XP_001841879.1 | *Culex quinquefasciatus* |
| Chitin synthase | TRINITY_DN37894_c0_g2 | 2.971559 | 6.020921 | -1.01876 | 8.74×10^-8^ | 6.15×10^-7^ | down | AKN90966.1 | *Macrobrachium nipponense* |
| Calcium-activated chloride channel | TRINITY_DN40369_c0_g1 | 116.9363 | 235.0676 | -1.00735 | 1.44×10^-29^ | 3.05×10^-28^ | down | KDR08671.1 | *Zootermopsis nevadensis* |
| Calcium-activated chloride channel | TRINITY_DN37980_c2_g1 | 9.196463 | 23.09478 | -1.32842 | 4.67×10^-42^ | 1.42×10^-40^ | down | - | *-* |
| Calcium-activated chloride channel | TRINITY_DN35481_c2_g1 | 1.384403 | 6.494136 | -2.22987 | 9.14×10^-25^ | 1.64×10^-23^ | down | EFX71591.1 | *Daphnia pulex* |
| Calcium-activated chloride channel | TRINITY_DN39887_c1_g2 | 8.728728 | 19.07341 | -1.12772 | 1.61×10^-18^ | 2.24×10^-17^ | down | - | *-* |
| Cytochrome oxidase assembly protein （ COX15-CtaA） | TRINITY_DN34481_c0_g1 | 12.42289 | 42.2866 | -1.7672 | 1.20×10^-3^ | 4.56×10^-3^ | down | XP_321341.3 | *Anopheles gambiae str. PEST* |
| Cytochrome oxidase assembly protein （ COX16） | TRINITY_DN36416_c0_g4 | 36.5457 | 17.0444 | 1.100404 | 6.21×10^-19^ | 8.83×10^-18^ | up | XP_002408093.1 | *Ixodes scapularis* |
| Cytochrome oxidase assembly protein （ COX4） | TRINITY_DN32699_c2_g8 | 213.558 | 84.95232 | 1.329903 | 9.56×10^-293^ | 2.47×10^-290^ | up | AFY10818.1 | *Litopenaeus vannamei* |
| Cytochrome oxidase assembly protein （COX6B） | TRINITY_DN31951_c0_g6 | 259.003 | 118.09 | 1.133082 | 5.28×10^-230^ | 1.06×10^-227^ | up | AIU99749.1 | *Litopenaeus vannamei* |
| Cytochrome oxidase assembly protein （COX6C） | TRINITY_DN32513_c0_g4 | 402.951 | 144.0535 | 1.483999 | 9.01×10^-182^ | 1.31×10^-179^ | up | AIU99750.1 | *Litopenaeus vannamei* |
| Catenin-beta-like | TRINITY_DN33336_c3_g1 | 7.89508 | 23.15072 | -1.55203 | 1.95×10^-33^ | 4.68×10^-32^ | down | XP_013791309.1 | *Limulus polyphemus* |
| Copper type II ascorbate-dependent monooxygenase | TRINITY_DN39237_c0_g1 | 8.004267 | 21.34971 | -1.41538 | 3.85×10^-19^ | 5.52×10^-18^ | down | KZS14172.1 | *Daphnia magna* |
| cullin5 | TRINITY_DN33761_c0_g3 | 6.004014 | 12.1186 | -1.01322 | 2.98×10^-40^ | 8.68×10^-39^ | down | AMJ52358.1 | *Eriocheir sinensis* |
| cullin2 | TRINITY_DN38091_c2_g1 | 14.43242 | 32.15759 | -1.15585 | 5.46×10^-32^ | 1.25×10^-30^ | down | AMJ52355.1 | *Eriocheir sinensis* |
| cullin1 | TRINITY_DN36695_c0_g4 | 6.576259 | 15.7121 | -1.25654 | 8.50×10^-19^ | 1.20×10^-17^ | down | AMJ52354.1 | *Eriocheir sinensis* |
| Cupin_4 | TRINITY_DN36822_c1_g1 | 18.7048 | 51.81934 | -1.47008 | 9.08×10^-88^ | 5.89×10^-86^ | down | XP_009061589.1 | *Lottia gigantea* |
| Cupin_8 | TRINITY_DN35784_c3_g1 | 59.8485 | 28.3732 | 1.076786 | 3.42×10^-75^ | 1.91×10^-73^ | up | XP_015461999.1 | *Astyanax mexicanus* |
| Cytochrome b | TRINITY_DN41227_c1_g1 | 840.5833 | 2256.793 | -1.42481 | 0 | 0 | down | YP_002650703.1 | *Macrobrachium lanchesteri* |
| E1-E2_ATPase | TRINITY_DN39120_c1_g7 | 48.7496 | 144.4266 | -1.56687 | 2.80×10^-219^ | 5.29×10^-217^ | down | AJO70000.1 | *Cherax destructor* |
| E1-E2_ATPase | TRINITY_DN40652_c1_g2 | 6.541249 | 15.71104 | -1.26414 | 1.17×10^-13^ | 1.27×10^-12^ | down | XP_015514166.1 | *Neodiprion lecontei* |
| E1-E2_ATPase | TRINITY_DN35641_c0_g4 | 10.02682 | 20.90743 | -1.06015 | 1.43×10^-10^ | 1.27×10^-9^ | down | AFE19188.1 | *Callinectes sapidus* |
| E3 ubiquitin-protein ligase UBR4 | TRINITY_DN40990_c2_g1 | 6.605314 | 14.34214 | -1.11856 | 8.78×10^-160^ | 1.11×10^-157^ | down | KFM56803.1 | *Stegodyphus mimosarum* |
| E3 ubiquitin-protein ligase | TRINITY_DN31459_c2_g4 | 1.821041 | 8.491675 | -2.22129 | 8.95×10^-24^ | 1.56×10^-22^ | down | XP_013774853.1 | *Limulus polyphemus* |
| Ecdysteroid kinase | TRINITY_DN37938_c1_g5 | 7.116522 | 14.28195 | -1.00495 | 4.85×10^-8^ | 3.50×10^-7^ | down | - | *-* |
| Ecdysteroid kinase | TRINITY_DN35268_c0_g3 | 2.45336 | 5.39558 | -1.13702 | 1.72×10^-5^ | 9.17×10^-5^ | down | KRT83353.1 | *Oryctes borbonicus* |
| Translation elongation factor | TRINITY_DN34629_c2_g1 | 409.8759 | 163.4195 | 1.326607 | 0 | 0 | up | AAS65797.1 | *Balanus glandula* |
| EF-hand domain | TRINITY_DN34118_c0_g1 | 14.66088 | 30.21054 | -1.04308 | 9.61×10^-38^ | 2.62×10^-36^ | down | XP_012175903.1 | *Bombus terrestris* |
| EF-hand domain | TRINITY_DN36446_c3_g1 | 9.508063 | 43.12589 | -2.18133 | 8.92×10^-215^ | 1.63×10^-212^ | down | KZS13220.1 | *Daphnia magna* |
| EF-hand domain | TRINITY_DN33865_c3_g2 | 4.528296 | 18.06625 | -1.99626 | 1.54×10^-60^ | 6.74×10^-59^ | down | KDR08227.1 | *Zootermopsis nevadensis* |
| EF-hand domain （Epidermal growth factor receptor） | TRINITY_DN40918_c0_g2 | 2.566939 | 10.82123 | -2.07574 | 1.04×10^-41^ | 3.11×10^-40^ | down | XP_012215593.1 | *Linepithema humile* |
| EF-hand domain | TRINITY_DN37947_c0_g1 | 9.755513 | 23.8357 | -1.28883 | 1.65×10^-52^ | 6.27×10^-51^ | down | XP_013794214.1 | *Limulus polyphemus* |
| EF-hand domain | TRINITY_DN25980_c0_g1 | 15.4672 | 0.0001 | 17.23885 | 1.40×10^-37^ | 3.79×10^-36^ | up | ACU00106.1 | *Homarus americanus* |
| FtsJ-like methyltransferase | TRINITY_DN34032_c3_g1 | 4.54044 | 15.1663 | -1.73997 | 2.63×10^-47^ | 8.91×10^-46^ | down | KYQ58625.1 | *Trachymyrmex zeteki* |
| FtsJ-like methyltransferase | TRINITY_DN32274_c2_g3 | 4.65028 | 9.521384 | -1.03385 | 5.71×10^-4^ | 2.32×10^-3^ | down | EFX74631.1 | *Daphnia pulex* |
| Ion channel | TRINITY_DN36762_c2_g1 | 907.0043 | 399.4598 | 1.183059 | 2.17×10^-111^ | 1.83×10^-109^ | up | XP_013771695.1 | *Limulus polyphemus* |
| Ion channel | TRINITY_DN31465_c0_g2 | 3.05072 | 9.140101 | -1.58306 | 1.80×10^-7^ | 1.22×10^-6^ | down | XP_013780582.1 | *Limulus polyphemus* |
| Ligand-gated ion channel | TRINITY_DN33126_c0_g3 | 1.979361 | 5.01789 | -1.34205 | 2.54×10^-20^ | 3.86×10^-19^ | down | - | *-* |
| Ligand-gated ion channel | TRINITY_DN29785_c0_g1 | 0.545493 | 5.758023 | -3.39994 | 3.26×10^-20^ | 4.93×10^-19^ | down | - | *-* |
| Ligand-gated ion channel | TRINITY_DN33121_c1_g1 | 15.09031 | 34.05575 | -1.17428 | 1.74×10^-19^ | 2.53×10^-18^ | down | XP_012341156.1 | *Apis florea* |
| Ligand-gated ion channel | TRINITY_DN34498_c0_g1 | 1.858455 | 6.068613 | -1.70726 | 7.50×10^-15^ | 8.68×10^-14^ | down | - | *-* |
| Ligand-gated ion channel | TRINITY_DN38314_c1_g1 | 2.739917 | 6.516321 | -1.24993 | 2.46×10^-5^ | 1.29×10^-4^ | down | - | *-* |
| Ligand-gated ion channel | TRINITY_DN41056_c7_g1 | 10.48091 | 25.14808 | -1.26268 | 4.94×10^-5^ | 2.45×10^-4^ | down | CEF34378.1 | *Coenobita clypeatus* |
| Ligand-gated ion channel | TRINITY_DN40351_c1_g6 | 860.3652 | 349.1582 | 1.301068 | 0 | 0 | up | XP_013779399.1 | *Limulus polyphemus* |
| Methyltransferase | TRINITY_DN39657_c2_g1 | 7.521271 | 18.92913 | -1.33156 | 5.30×10^-74^ | 2.90×10^-72^ | down | XP_014666776.1 | *Priapulus caudatus* |
| Methyltransferase | TRINITY_DN30269_c0_g1 | 5.362853 | 11.2071 | -1.06334 | 1.20×10^-9^ | 9.95×10^-9^ | down | ELU08967.1 | *Capitella teleta* |
| Methyltransferase | TRINITY_DN34371_c0_g1 | 2.214443 | 10.30189 | -2.21789 | 3.07×10^-30^ | 6.64×10^-29^ | down | AEE61646.1 | *Dendroctonus ponderosae* |
| Methyltransferase | TRINITY_DN36282_c2_g1 | 2.66666 | 5.575559 | -1.06408 | 2.08×10^-5^ | 1.10×10^-4^ | down | XP_015172725.1 | *Polistes dominula* |
| Sodium / potassium ATPase | TRINITY_DN37337_c1_g1 | 2.757942 | 15.39699 | -2.48098 | 5.73×10^-75^ | 3.18×10^-73^ | down | XP_011675407.1 | *Strongylocentrotus purpuratus* |
| Sodium / potassium ATPase | TRINITY_DN37181_c1_g1 | 5.09203 | 16.71735 | -1.71503 | 3.22×10^-17^ | 4.21×10^-16^ | down | AEE25938.1 | *Litopenaeus vannamei* |
| Neurotransmitter-gated ion-channel ligand binding domain | TRINITY_DN30344_c3_g1 | 29.09431 | 65.66213 | -1.17432 | 3.44×10^-8^ | 2.51×10^-7^ | down | XP_015367853.1 | *Diuraphis noxia* |
| Neurotransmitter-gated ion-channel ligand binding domain | TRINITY_DN30141_c1_g1 | 3.538599 | 7.849651 | -1.14945 | 1.47×10^-2^ | 4.03×10^-2^ | down | P91766.1 | *-* |
| Cytochrome P450 | TRINITY_DN40506_c0_g2 | 103.2572 | 230.366 | -1.15769 | 2.49×10^-211^ | 4.48×10^-209^ | down | AJA06113.1 | *Portunus trituberculatus* |
| Renin receptor | TRINITY_DN33692_c2_g2 | 19.81313 | 47.90709 | -1.27378 | 1.91×10^-33^ | 4.58×10^-32^ | down | XP_973593.1 | *Tribolium castaneum* |
| Ion channel regulatory protein | TRINITY_DN31467_c4_g2 | 0.489069 | 11.59297 | -4.56707 | 4.64×10^-8^ | 3.34×10^-7^ | down | XP_014290343.1 | *Halyomorpha halys* |
| Vitellogenin | TRINITY_DN39032_c0_g3 | 3.18601 | 14.3085 | -2.16705 | 1.82×10^-124^ | 1.74×10^-122^ | down | AHA85992.1 | *Macrobrachium rosenbergii* |
| Voltage gated chloride channel | TRINITY_DN35816_c0_g2 | 4.260831 | 10.56241 | -1.30973 | 6.76×10^-39^ | 1.89×10^-37^ | down | XP_011150001.1 | *Harpegnathos saltator* |
| Ankyrin repeats | TRINITY_DN38589_c1_g1 | 24.59741 | 96.37782 | -1.9702 | 0 | 0 | down | KDR06504.1 | *Zootermopsis nevadensis* |
| Ankyrin repeats | TRINITY_DN36896_c2_g2 | 43.90615 | 115.2387 | -1.39213 | 1.70×10^-307^ | 4.69×10^-305^ | down | XP_013783021.1 | *Limulus polyphemus* |
| Ankyrin repeats | TRINITY_DN32819_c1_g5 | 10.7912 | 43.16468 | -2 | 2.54×10^-154^ | 3.08×10^-152^ | down | KDR06504.1 | *Zootermopsis nevadensis* |
| Ankyrin repeats | TRINITY_DN40634_c3_g1 | 29.83487 | 99.96818 | -1.74447 | 1.06×10^-123^ | 1.00×10^-121^ | down | KDR21363.1 | *Zootermopsis nevadensis* |
| Ankyrin repeats | TRINITY_DN40777_c2_g1 | 1.25573 | 6.71451 | -2.41876 | 2.47×10^-90^ | 1.65×10^-88^ | down | ABK56706.1 | *Parhyale hawaiensis* |
| Ankyrin repeats | TRINITY_DN38276_c0_g2 | 10.5101 | 31.76155 | -1.59551 | 1.91×10^-73^ | 1.03×10^-71^ | down | XP_014261425.1 | *Cimex lectularius* |
| Ankyrin repeats | TRINITY_DN37526_c1_g2 | 2.672339 | 9.708921 | -1.86121 | 2.02×10^-55^ | 8.06×10^-54^ | down | XP_015509201.1 | *Neodiprion lecontei* |
| Ankyrin repeats | TRINITY_DN36263_c1_g1 | 14.33672 | 28.84702 | -1.00871 | 1.79×10^-39^ | 5.12×10^-38^ | down | XP_014467230.1 | *Dinoponera quadriceps* |
| Ankyrin repeats | TRINITY_DN41013_c5_g2 | 6.86855 | 16.5015 | -1.26452 | 8.14×10^-38^ | 2.23×10^-36^ | down | XP_015763923.1 | *Acropora digitifera* |
| Ankyrin repeats | TRINITY_DN32589_c0_g1 | 1.317073 | 5.127444 | -1.9609 | 2.69×10^-37^ | 7.24×10^-36^ | down | KDR20941.1 | *Zootermopsis nevadensis* |
| Ankyrin repeats | TRINITY_DN38213_c2_g1 | 2.593285 | 5.80015 | -1.16131 | 1.36×10^-33^ | 3.29×10^-32^ | down | KFM81130.1 | *Stegodyphus mimosarum* |
| Ankyrin repeats | TRINITY_DN35900_c1_g1 | 5.003545 | 14.43693 | -1.52874 | 4.08×10^-33^ | 9.68×10^-32^ | down | XP_015373513.1 | *Diuraphis noxia* |
| Ankyrin repeats | TRINITY_DN36836_c0_g1 | 8.5576 | 19.9511 | -1.22119 | 4.81×10^-33^ | 1.14×10^-31^ | down | XP_013381147.1 | *Lingula anatina* |
| Ankyrin repeats (NF-kappa B inhibitor alpha) | TRINITY_DN36427_c1_g4 | 1.576066 | 8.535239 | -2.4371 | 1.75×10^-32^ | 4.08×10^-31^ | down | AET34918.1 | *Macrobrachium rosenbergii* |
| Ankyrin repeats | TRINITY_DN40910_c6_g1 | 2.67772 | 12.43438 | -2.21526 | 7.41×10^-32^ | 1.69×10^-30^ | down | KDR14974.1 | *Zootermopsis nevadensis* |
| Ankyrin repeats (NF-kappa B inhibitor alpha) | TRINITY_DN34108_c0_g3 | 2.631019 | 6.97142 | -1.40583 | 7.17×10^-25^ | 1.29×10^-23^ | down | KDR17553.1 | *Zootermopsis nevadensis* |
| Ankyrin repeats | TRINITY_DN39150_c2_g1 | 4.280321 | 9.8937 | -1.20879 | 1.78×10^-22^ | 2.96×10^-21^ | down | XP_013089649.1 | *Biomphalaria glabrata* |
| Ankyrin repeats (E3 ubiquitin-protein ligase) | TRINITY_DN39383_c1_g2 | 11.41186 | 28.35302 | -1.31297 | 9.50×10^-17^ | 1.21×10^-15^ | down | XP_013774252.1 | *Limulus polyphemus* |
| Ankyrin repeats | TRINITY_DN36707_c1_g3 | 2.68195 | 5.394844 | -1.0083 | 3.45×10^-10^ | 3.00×10^-9^ | down | - | *-* |
| Ankyrin repeats | TRINITY_DN37394_c0_g1 | 2.022753 | 5.392961 | -1.41476 | 2.23×10^-8^ | 1.65×10^-7^ | down | - | *-* |
| Ankyrin repeats | TRINITY_DN33349_c0_g3 | 2.059757 | 5.756624 | -1.48275 | 1.26×10^-7^ | 8.70×10^-7^ | down | KPP77962.1 | *Scleropages formosus* |
| Ankyrin repeats | TRINITY_DN40398_c2_g2 | 1.91816 | 7.59463 | -1.98526 | 6.88×10^-7^ | 4.39×10^-6^ | down | XP_010595571.1 | *Loxodonta africana* |
| Ankyrin repeats | TRINITY_DN34487_c0_g1 | 2.350647 | 5.844747 | -1.31408 | 1.39×10^-6^ | 8.58×10^-6^ | down | XP_005798131.1 | *Xiphophorus maculatus* |
| Ankyrin repeats | TRINITY_DN33387_c1_g2 | 2.544904 | 5.73744 | -1.1728 | 3.09×10^-4^ | 1.33×10^-3^ | down | XP_015595700.1 | *Cephus cinctus* |
| Ankyrin repeats | TRINITY_DN31871_c1_g1 | 3.02367 | 7.20622 | -1.25294 | 3.25×10^-4^ | 1.40×10^-3^ | down | XP_013783602.1 | *Limulus polyphemus* |
| Ankyrin repeats | TRINITY_DN36961_c0_g1 | 2.006821 | 6.631203 | -1.72436 | 2.26×10^-3^ | 8.02×10^-3^ | down | XP_015518771.1 | *Neodiprion lecontei* |
| Ubiquitin-like autophagy protein Apg12 | TRINITY_DN34281_c0_g3 | 28.18855 | 13.0801 | 1.107736 | 1.39×10^-30^ | 3.04×10^-29^ | up | EHJ78946.1 | *Danaus plexippus* |
| Autophagy protein Atg8 ubiquitin | TRINITY_DN39758_c1_g6 | 127.9731 | 425.0816 | -1.7319 | 0 | 0 | down | KDR08012.1 | *Zootermopsis nevadensis* |
| Autophagy protein Atg8 ubiquitin | TRINITY_DN31900_c0_g1 | 47.87102 | 99.45624 | -1.05491 | 1.21×10^-66^ | 5.95×10^-65^ | down | XP_312238.3 | *Anopheles gambiae str. PEST* |
| Autophagy protein Atg8 ubiquitin | TRINITY_DN36752_c1_g1 | 12.34207 | 29.98056 | -1.28044 | 8.15×10^-40^ | 2.35×10^-38^ | down | - | *-* |
| Clathrin adaptor | TRINITY_DN34056_c1_g2 | 7.02621 | 19.1687 | -1.44793 | 4.99×10^-38^ | 1.37×10^-36^ | down | AHE63353.1 | *Penaeus monodon* |
| EGF | TRINITY_DN36163_c1_g3 | 1.156922 | 5.654672 | -2.28915 | 1.69×10^-23^ | 2.90×10^-22^ | down | XP_008196296.1 | *Tribolium castaneum* |
| EGF | TRINITY_DN40430_c1_g1 | 6.121737 | 24.38146 | -1.99377 | 7.73×10^-229^ | 1.54×10^-226^ | down | XP_015750954.1 | *Acropora digitifera* |
| Hormone receptor | TRINITY_DN39424_c0_g5 | 7.24921 | 19.4442 | -1.42344 | 2.77×10^-17^ | 3.65×10^-16^ | down | XP_007655145.1 | *Ornithorhynchus anatinus* |
| Hsp70 | TRINITY_DN34485_c1_g2 | 172.9753 | 76.96378 | 1.168315 | 1.20×10^-296^ | 3.21×10^-294^ | up | AKB96215.1 | *Cherax cainii* |
| Hsp70 | TRINITY_DN34354_c1_g1 | 18.0711 | 51.76042 | -1.51816 | 8.82×10^-73^ | 4.72×10^-71^ | down | AKB96210.1 | *Cherax destructor* |
| Hsp90 | TRINITY_DN41123_c1_g1 | 382.6259 | 827.9927 | -1.11368 | 0 | 0 | down | AGC54636.1 | *Scylla paramamosain* |
| Hsp90 | TRINITY_DN38649_c1_g1 | 30.35417 | 78.71452 | -1.37474 | 3.90×10^-36^ | 1.02×10^-34^ | down | AKB96203.1 | *Cherax quadricarinatus* |
| Low-density lipoprotein receptor | TRINITY_DN40884_c2_g3 | 52.51756 | 113.8316 | -1.11603 | 0 | 0 | down | ADK55596.1 | *Macrobrachium rosenbergii* |
| Low-density lipoprotein receptor | TRINITY_DN40799_c1_g1 | 1.255814 | 11.33217 | -3.17373 | 1.29×10^-105^ | 1.04×10^-103^ | down | XP_014044934.1 | *Salmo salar* |
| Low-density lipoprotein receptor | TRINITY_DN36471_c0_g1 | 20.51641 | 41.6789 | -1.02254 | 7.18×10^-28^ | 1.43×10^-26^ | down | XP_005111446.2 | *Aplysia californica* |
| Low-density lipoprotein receptor | TRINITY_DN40346_c0_g2 | 2.918309 | 11.43458 | -1.9702 | 8.00×10^-21^ | 1.24×10^-19^ | down | XP_013772237.1 | *Limulus polyphemus* |
| Low-density lipoprotein receptor | TRINITY_DN31703_c0_g1 | 8.535338 | 18.62132 | -1.12544 | 1.55×10^-2^ | 4.22×10^-2^ | down | AHL26189.1 | *Pandalopsis japonica* |
| Phosphoenolpyruvate carboxykinase | TRINITY_DN32884_c0_g1 | 21.73126 | 61.57332 | -1.50253 | 3.53×10^-77^ | 2.03×10^-75^ | down | ALK82313.1 | *Macrobrachium nipponense* |
| Phosphoenolpyruvate carboxykinase | TRINITY_DN39916_c2_g2 | 2.65158 | 6.40007 | -1.27124 | 3.69×10^-4^ | 1.56×10^-3^ | down | ALK82313.1 | *Macrobrachium nipponense* |
| Peptidase_A17 | TRINITY_DN41181_c5_g1 | 13.66578 | 77.90628 | -2.51117 | 0 | 0 | down | XP_006811861.1 | *Saccoglossus kowalevskii* |
| Peptidase_A18 | TRINITY_DN40926_c0_g1 | 2.16534 | 13.69062 | -2.66052 | 8.08×10^-93^ | 5.59×10^-91^ | down | XP_011436930.1 | *Crassostrea gigas* |
| Peptidase_A19 | TRINITY_DN40632_c3_g1 | 3.062561 | 13.98381 | -2.19095 | 2.80×10^-52^ | 1.06×10^-50^ | down | - | *-* |
| Peptidase_A20 | TRINITY_DN34444_c0_g2 | 0.560943 | 6.679025 | -3.57371 | 4.02×10^-10^ | 3.47×10^-9^ | down | - | *-* |
| Peptidase_A21 | TRINITY_DN40406_c0_g1 | 0.259818 | 9.509435 | -5.19379 | 1.43×10^-9^ | 1.18×10^-8^ | down | - | *-* |
| Peptidase_A22 | TRINITY_DN35871_c0_g1 | 0.490274 | 5.028303 | -3.35841 | 1.71×10^-8^ | 1.29×10^-7^ | down | XP_006817263.1 | *Saccoglossus kowalevskii* |
| Peptidase_A23 | TRINITY_DN30980_c3_g1 | 0.643668 | 6.999585 | -3.44288 | 4.57×10^-7^ | 2.98×10^-6^ | down | KHN88744.1 | *Toxocara canis* |
| Peptidase_A24 (cathepsinL) | TRINITY_DN41160_c1_g1 | 225.6163 | 469.8838 | -1.05843 | 8.09×10^-53^ | 3.10×10^-51^ | down | AGN52717.1 | *Macrobrachium rosenbergii* |
| Peptidase_A25 | TRINITY_DN34643_c1_g2 | 1.087449 | 11.02968 | -3.34237 | 1.42×10^-35^ | 3.65×10^-34^ | down | KZS14202.1 | *Daphnia magna* |
| Peptidase_A26 | TRINITY_DN39561_c0_g2 | 6.692733 | 16.98938 | -1.34397 | 9.87×10^-21^ | 1.52×10^-19^ | down | XP_011434325.1 | *Crassostrea gigas* |
| Peptidase_A27 (cathepsinC) | TRINITY_DN38629_c0_g6 | 2.881009 | 5.76279 | -1.00019 | 1.91×10^-4^ | 8.56×10^-4^ | down | ACK57788.1 | *Litopenaeus vannamei* |
| Peptidase_A28 | TRINITY_DN31027_c1_g1 | 7.612131 | 15.62395 | -1.03739 | 1.85×10^-23^ | 3.19×10^-22^ | down | XP_015189134.1 | *Polistes dominula* |
| Peptidase_A29 (caspase3C) | TRINITY_DN37408_c0_g2 | 10.09712 | 33.57045 | -1.73325 | 3.56×10^-103^ | 2.81×10^-101^ | down | AET34920.1 | *Macrobrachium rosenbergii* |
| Peptidase_A30 (caspase) | TRINITY_DN36074_c0_g1 | 1.785705 | 7.765348 | -2.12056 | 8.24×10^-18^ | 1.11×10^-16^ | down | ADM45311.1 | *Eriocheir sinensis* |
| Peptidase_A31 | TRINITY_DN38799_c1_g1 | 10.6068 | 32.6201 | -1.62077 | 2.79×10^-37^ | 7.50×10^-36^ | down | AAT77811.1 | *Gecarcinus lateralis* |
| Peptidase_A32 | TRINITY_DN33556_c0_g1 | 6.635432 | 17.06799 | -1.36303 | 1.41×10^-7^ | 9.70×10^-7^ | down | KYN01745.1 | *Cyphomyrmex costatus* |
| Peptidase_A33 | TRINITY_DN36032_c1_g4 | 5.410609 | 17.90942 | -1.72686 | 3.99×10^-68^ | 2.01×10^-66^ | down | XP_013794807.1 | *Limulus polyphemus* |
| Peptidase_A34 | TRINITY_DN35133_c1_g2 | 6.649373 | 18.93737 | -1.50995 | 6.61×10^-62^ | 2.97×10^-60^ | down | XP_015586243.1 | *Cephus cinctus* |
| Peptidase_A35 | TRINITY_DN36792_c0_g1 | 7.184788 | 15.08717 | -1.0703 | 6.00×10^-18^ | 8.14×10^-17^ | down | XP_015109305.1 | *Diachasma alloeum* |
| Peptidase_A36 | TRINITY_DN38703_c0_g1 | 13.41366 | 31.01789 | -1.2094 | 1.96×10^-11^ | 1.85×10^-10^ | down | XP_015793163.1 | *Tetranychus urticae* |
| Peptidase_A37 | TRINITY_DN36556_c1_g1 | 3.857156 | 8.035645 | -1.05888 | 2.08×10^-32^ | 4.83×10^-31^ | down | XP_015184599.1 | *Polistes dominula* |
| Peptidase_A38 | TRINITY_DN33446_c1_g1 | 1.802964 | 6.67766 | -1.88897 | 1.04×10^-36^ | 2.75×10^-35^ | down | EFX80961.1 | *Daphnia pulex* |
| Peptidase_A39 | TRINITY_DN31105_c2_g6 | 1.10566 | 6.43386 | -2.54078 | 1.05×10^-18^ | 1.47×10^-17^ | down | - | *-* |
| Peptidase_A40 | TRINITY_DN38302_c0_g1 | 2.224075 | 6.931303 | -1.63992 | 3.51×10^-12^ | 3.49×10^-11^ | down | KDR19260.1 | *Zootermopsis nevadensis* |
| Peptidase_A41 | TRINITY_DN38095_c0_g4 | 23.32459 | 69.27066 | -1.57039 | 1.27×10^-144^ | 1.40×10^-142^ | down | XP_015114563.1 | *Diachasma alloeum* |
| Peptidase_A42 | TRINITY_DN37017_c0_g1 | 28.22527 | 58.47826 | -1.05091 | 9.99×10^-52^ | 3.72×10^-50^ | down | XP_006007579.1 | *Latimeria chalumnae* |
| Peptidase_A43 | TRINITY_DN33546_c0_g6 | 19.22881 | 41.8234 | -1.12104 | 1.51×10^-52^ | 5.74×10^-51^ | down | XP_014246481.1 | *Cimex lectularius* |
| Peptidase_A44 | TRINITY_DN35962_c0_g1 | 10.91148 | 22.88021 | -1.06825 | 5.66×10^-26^ | 1.07×10^-24^ | down | XP_014671353.1 | *Priapulus caudatus* |
| Peptidase_A45 | TRINITY_DN32288_c4_g2 | 5.205825 | 11.6244 | -1.15896 | 1.89×10^-18^ | 2.62×10^-17^ | down | KDR07700.1 | *Zootermopsis nevadensis* |
| Peptidase_A46 | TRINITY_DN38751_c1_g1 | 53.82142 | 108.0784 | -1.00583 | 6.09×10^-85^ | 3.81×10^-83^ | down | XP_008549924.1 | *Microplitis demolitor* |
| Peptidase_A47 (cathepsinA) | TRINITY_DN36472_c0_g1 | 10.03235 | 25.2148 | -1.32961 | 1.80×10^-52^ | 6.85×10^-51^ | down | ADO65982.1 | *Eriocheir sinensis* |
| Peptidase_A48 | TRINITY_DN39647_c1_g2 | 13.21927 | 44.40211 | -1.74799 | 7.93×10^-50^ | 2.83×10^-48^ | down | ACO11096.1 | *Caligus rogercresseyi* |
| Peptidase_A49 | TRINITY_DN37555_c0_g1 | 5.510617 | 15.00947 | -1.44559 | 5.64×10^-37^ | 1.50×10^-35^ | down | XP_013778463.1 | *Limulus polyphemus* |
| Peptidase_A50 | TRINITY_DN31163_c0_g5 | 1.550251 | 5.10575 | -1.71962 | 4.86×10^-7^ | 3.15×10^-6^ | down | XP_014280468.1 | *Halyomorpha halys* |
| Peptidase_A51 | TRINITY_DN35533_c0_g1 | 4.3051 | 10.66974 | -1.30941 | 6.51×10^-22^ | 1.06×10^-20^ | down | KDR12950.1 | *Zootermopsis nevadensis* |
| Peptidase_A52 | TRINITY_DN33424_c1_g1 | 1.502247 | 5.94179 | -1.98378 | 4.61×10^-8^ | 3.32×10^-7^ | down | XP_011690516.1 | *Wasmannia auropunctata* |
| Protein tyrosine kinase (insulin-like growth factor 1 receptor) | TRINITY_DN40945_c4_g1 | 4.760539 | 15.58348 | -1.71082 | 3.78×10^-109^ | 3.13×10^-107^ | down | XP_015809442.1 | *Nothobranchius furzeri* |
| Protein tyrosine kinase | TRINITY_DN36076_c0_g1 | 11.7475 | 38.74985 | -1.72184 | 1.25×10^-78^ | 7.33×10^-77^ | down | - | *-* |
| Protein tyrosine kinase (insulin Receptor) | TRINITY_DN39910_c0_g1 | 4.445347 | 13.26597 | -1.57736 | 1.12×10^-75^ | 6.26×10^-74^ | down | CDI30232.1 | *Blattella germanica* |
| Protein tyrosine kinase | TRINITY_DN38502_c2_g1 | 3.845132 | 14.43843 | -1.90881 | 1.39×10^-58^ | 5.92×10^-57^ | down | EFX90330.1 | *Daphnia pulex* |
| Protein tyrosine kinase | TRINITY_DN37931_c0_g1 | 2.923417 | 10.41634 | -1.83312 | 8.82×10^-56^ | 3.55×10^-54^ | down | XP_013784087.1 | *Limulus polyphemus* |
| Protein tyrosine kinase | TRINITY_DN36604_c0_g1 | 1.475059 | 7.043197 | -2.25546 | 1.08×10^-39^ | 3.09×10^-38^ | down | KZS15911.1 | *Daphnia magna* |
| Protein tyrosine kinase | TRINITY_DN39848_c1_g2 | 8.44813 | 25.37471 | -1.58669 | 7.98×10^-37^ | 2.12×10^-35^ | down | KDR09664.1 | *Zootermopsis nevadensis* |
| Protein tyrosine kinase | TRINITY_DN34294_c2_g1 | 3.14048 | 8.329976 | -1.40733 | 4.79×10^-31^ | 1.06×10^-29^ | down | XP_015114804.1 | *Diachasma alloeum* |
| Protein tyrosine kinase | TRINITY_DN31498_c0_g1 | 1.25737 | 5.191652 | -2.04578 | 2.91×10^-19^ | 4.20×10^-18^ | down | XP_013774329.1 | *Limulus polyphemus* |
| Protein tyrosine kinase | TRINITY_DN39854_c0_g4 | 5.32099 | 11.463 | -1.10722 | 1.16×10^-17^ | 1.55×10^-16^ | down | XP_013776236.1 | *Limulus polyphemus* |
| Protein tyrosine kinase | TRINITY_DN33309_c0_g1 | 10.02792 | 20.9865 | -1.06544 | 1.56×10^-13^ | 1.68×10^-12^ | down | XP_013782545.1 | *Limulus polyphemus* |
| Protein tyrosine kinase | TRINITY_DN32265_c1_g4 | 3.683899 | 13.97987 | -1.92405 | 1.09×10^-12^ | 1.11×10^-11^ | down | XP_014601948.1 | *Polistes canadensis* |
| Protein tyrosine kinase | TRINITY_DN30344_c3_g2 | 4.799165 | 11.5908 | -1.27212 | 2.38×10^-10^ | 2.09×10^-9^ | down | EFX65169.1 | *Daphnia pulex* |
| Protein tyrosine kinase | TRINITY_DN32651_c4_g1 | 2.715313 | 7.415605 | -1.44945 | 2.41×10^-8^ | 1.78×10^-7^ | down | KFM75830.1 | *Stegodyphus mimosarum* |
| Protein tyrosine kinase | TRINITY_DN36078_c1_g1 | 4.005434 | 11.54075 | -1.52671 | 3.31×10^-6^ | 1.95×10^-5^ | down | KXJ83168.1 | *Aedes albopictus* |
| Protein tyrosine kinase | TRINITY_DN34223_c1_g2 | 3.82635 | 10.81166 | -1.49855 | 6.96×10^-6^ | 3.93×10^-5^ | down | XP_002424340.1 | *Pediculus humanus corporis* |
| Protein tyrosine kinase | TRINITY_DN37716_c0_g1 | 169.1925 | 11.33589 | 3.899696 | 1.72×10^-4^ | 7.79×10^-4^ | up | KDR20715.1 | *Zootermopsis nevadensis* |
| Trypsin | TRINITY_DN39872_c0_g1 | 90.76306 | 282.4371 | -1.63775 | 0 | 0 | down | XP_015790479.1 | *Tetranychus urticae* |
| Trypsin | TRINITY_DN40958_c0_g1 | 52.69099 | 125.2406 | -1.24907 | 1.56×10^-259^ | 3.55×10^-257^ | down | - | *-* |
| Trypsin | TRINITY_DN33872_c1_g5 | 6.26632 | 24.24699 | -1.95212 | 4.58×10^-75^ | 2.55×10^-73^ | down | ACY66494.3 | *Scylla paramamosain* |
| Trypsin | TRINITY_DN37379_c3_g2 | 84.53947 | 175.9805 | -1.05772 | 5.14×10^-73^ | 2.77×10^-71^ | down | AJT34991.1 | *Procambarus clarkii* |
| Trypsin | TRINITY_DN34696_c0_g4 | 19.61374 | 45.52894 | -1.21492 | 6.59×10^-46^ | 2.17×10^-44^ | down | - | *-* |
| Trypsin | TRINITY_DN40457_c1_g1 | 10.0749 | 25.09542 | -1.31666 | 1.57×10^-38^ | 4.36×10^-37^ | down | EFX71934.1 | *Daphnia pulex* |
| Trypsin | TRINITY_DN39988_c0_g3 | 5.62139 | 21.6906 | -1.94807 | 8.06×10^-33^ | 1.90×10^-31^ | down | XP_011866109.1 | *Vollenhovia emeryi* |
| Trypsin | TRINITY_DN35774_c0_g1 | 2.410185 | 8.503703 | -1.81895 | 9.83×10^-29^ | 2.03×10^-27^ | down | CCW43203.1 | *Scylla paramamosain* |
| Trypsin | TRINITY_DN32324_c0_g4 | 0.986679 | 5.69222 | -2.52834 | 3.26×10^-14^ | 3.65×10^-13^ | down | CCW43203.1 | *Scylla paramamosain* |
| Trypsin | TRINITY_DN33395_c0_g3 | 2.899382 | 7.602749 | -1.39078 | 9.09×10^-14^ | 9.89×10^-13^ | down | XP_015379949.1 | *Diuraphis noxia* |
| Trypsin | TRINITY_DN36551_c0_g2 | 9.853973 | 3.81352 | 1.369582 | 1.94×10^-12^ | 1.96×10^-11^ | up | ADB66714.1 | *Panulirus argus* |
| Trypsin | TRINITY_DN37310_c2_g2 | 1.749481 | 5.264421 | -1.58935 | 1.56×10^-8^ | 1.18×10^-7^ | down | XP_002423000.1 | *Pediculus humanus corporis* |
| Trypsin | TRINITY_DN36944_c1_g1 | 13.0913 | 0.0001 | 16.99825 | 7.87×10^-8^ | 5.56×10^-7^ | up | XP_013809534.1 | *Apteryx australis mantelli* |
| Trypsin | TRINITY_DN31100_c1_g1 | 5.724421 | 18.13653 | -1.6637 | 4.34×10^-6^ | 2.51×10^-5^ | down | XP_006818053.1 | *Saccoglossus kowalevskii* |
| Trypsin | TRINITY_DN35943_c0_g4 | 2.57051 | 11.3192 | -2.13865 | 2.47×10^-5^ | 1.29×10^-4^ | down | ABO33174.1 | *Penaeus monodon* |
| Trypsin | TRINITY_DN30855_c0_g1 | 8.643572 | 17.55527 | -1.0222 | 3.15×10^-3^ | 1.06×10^-2^ | down | AAD21841.1 | *Ctenocephalides felis* |
| Trypsin | TRINITY_DN39125_c1_g3 | 2.179683 | 5.051696 | -1.21265 | 1.41×10^-2^ | 3.91×10^-2^ | down | AAD00320.1 | *Scolopendra subspinipes* |
| Ubiquinol-cytochrome C chaperone | TRINITY_DN37433_c0_g1 | 8.49086 | 19.45133 | -1.19589 | 6.52×10^-8^ | 4.64×10^-7^ | down | XP_308162.3 | *Anopheles gambiae str. PEST* |
| Ubiquitin | TRINITY_DN38763_c2_g1 | 822.7317 | 305.9575 | 1.427091 | 0 | 0 | up | AEB54653.1 | *Procambarus clarkii* |
| Ubiquitin | TRINITY_DN37222_c0_g1 | 293.9885 | 905.2497 | -1.62256 | 1.99×10^-210^ | 3.57×10^-208^ | down | XP_014867795.1 | *Poecilia mexicana* |
| Ubiquitin | TRINITY_DN36525_c2_g2 | 4.014163 | 11.63571 | -1.53539 | 1.31×10^-11^ | 1.25×10^-10^ | down | EFX62918.1 | *Daphnia pulex* |
| Ubiquitin carboxyl-terminal hydrolase | TRINITY_DN36764_c1_g3 | 12.12085 | 48.45047 | -1.99902 | 1.17×10^-230^ | 2.36×10^-228^ | down | XP_013409690.1 | *Lingula anatina* |
| Ubiquitin carboxyl-terminal hydrolase | TRINITY_DN37744_c0_g1 | 13.00179 | 31.79508 | -1.29009 | 4.07×10^-113^ | 3.49×10^-111^ | down | XP_011453343.1 | *Crassostrea gigas* |
| Ubiquitin carboxyl-terminal hydrolase | TRINITY_DN37243_c1_g2 | 15.36187 | 36.06723 | -1.23133 | 1.33×10^-46^ | 4.43×10^-45^ | down | XP_002429221.1 | *Pediculus humanus corporis* |
| Ubiquitin carboxyl-terminal hydrolase | TRINITY_DN33970_c0_g1 | 6.632094 | 14.93705 | -1.17136 | 1.10×10^-30^ | 2.41×10^-29^ | down | XP_013783746.1 | *Limulus polyphemus* |
| Ubiquitin carboxyl-terminal hydrolase | TRINITY_DN36947_c1_g2 | 1.829345 | 8.433491 | -2.2048 | 1.41×10^-27^ | 2.79×10^-26^ | down | XP_013394905.1 | *Lingula anatina* |
| Ubiquitin carboxyl-terminal hydrolase | TRINITY_DN38331_c3_g1 | 2.604724 | 8.298453 | -1.67171 | 4.63×10^-23^ | 7.89×10^-22^ | down | KDR20232.1 | *Zootermopsis nevadensis* |
| Ubiquitin carboxyl-terminal hydrolase | TRINITY_DN36847_c1_g1 | 2.649471 | 7.484465 | -1.49819 | 9.17×10^-23^ | 1.55×10^-21^ | down | KDR20232.1 | *Zootermopsis nevadensis* |
| Ubiquitin carboxyl-terminal hydrolase | TRINITY_DN35363_c2_g2 | 3.830869 | 15.30323 | -1.99809 | 2.52×10^-12^ | 2.53×10^-11^ | down | XP_002432627.1 | *Pediculus humanus corporis* |
| Ubiquitin carboxyl-terminal hydrolase | TRINITY_DN35415_c2_g2 | 5.76703 | 12.26952 | -1.08918 | 3.46×10^-11^ | 3.22×10^-10^ | down | XP_015518592.1 | *Neodiprion lecontei* |
| Ubiquitin carboxyl-terminal hydrolase | TRINITY_DN35177_c1_g1 | 2.58131 | 10.31596 | -1.9987 | 9.18×10^-4^ | 3.57×10^-3^ | down | XP_013074244.1 | *Biomphalaria glabrata* |
| Ubiquitin carboxyl-terminal hydrolase | TRINITY_DN35582_c1_g2 | 5.163928 | 11.54244 | -1.16041 | 2.80×10^-7^ | 1.86×10^-6^ | down | KOC66794.1 | *Habropoda laboriosa* |
| Ubiquitin elongating factor core | TRINITY_DN34056_c0_g1 | 5.2792 | 12.6766 | -1.26378 | 1.98×10^-31^ | 4.46×10^-30^ | down | XP_012285979.1 | *Orussus abietinus* |
| Ubiquitin elongating factor core | TRINITY_DN38510_c1_g1 | 2.461801 | 6.908499 | -1.48866 | 5.27×10^-10^ | 4.50×10^-9^ | down | KDR11245.1 | *Zootermopsis nevadensis* |
| Ubiquitin-conjugating enzyme | TRINITY_DN31635_c2_g2 | 85.8044 | 203.3226 | -1.24465 | 5.61×10^-159^ | 7.05×10^-157^ | down | KZC04983.1 | *Dufourea novaeangliae* |
| Ubiquitin-conjugating enzyme | TRINITY_DN40051_c1_g2 | 14.63264 | 30.89225 | -1.07805 | 3.89×10^-92^ | 2.66×10^-90^ | down | XP_012255958.1 | *Athalia rosae* |
| Ubiquitin-conjugating enzyme | TRINITY_DN33848_c1_g1 | 26.0198 | 64.63395 | -1.31268 | 1.10×10^-76^ | 6.25×10^-75^ | down | ACD13594.1 | *Penaeus monodon* |
| Ubiquitin-conjugating enzyme | TRINITY_DN39014_c1_g1 | 1.812701 | 6.279073 | -1.79241 | 1.96×10^-33^ | 4.70×10^-32^ | down | XP_013772644.1 | *Limulus polyphemus* |
| Ubiquitin-conjugating enzyme | TRINITY_DN33725_c1_g1 | 20.20226 | 42.4164 | -1.07011 | 7.58×10^-30^ | 1.62×10^-28^ | down | XP_002412813.1 | *Ixodes scapularis* |
| Ubiquitin-conjugating enzyme | TRINITY_DN40195_c3_g1 | 19.54967 | 39.75125 | -1.02386 | 1.40×10^-26^ | 2.68×10^-25^ | down | XP_013411458.1 | *Lingula anatina* |
| Ubiquitin-conjugating enzyme | TRINITY_DN39181_c2_g1 | 2.767133 | 6.073758 | -1.1342 | 3.16×10^-6^ | 1.87×10^-5^ | down | KDR09067.1 | *Zootermopsis nevadensis* |
| Forkhead-associated domain | TRINITY_DN33022_c0_g1 | 0.764573 | 7.930252 | -3.37464 | 6.62×10^-177^ | 9.42×10^-175^ | down | - | *-* |
| Forkhead | TRINITY_DN31656_c2_g2 | 18.02086 | 41.7603 | -1.21246 | 1.83×10^-56^ | 7.47×10^-55^ | down | XP_011268706.1 | *Camponotus floridanus* |
| Forkhead | TRINITY_DN38199_c1_g2 | 7.153303 | 15.63797 | -1.12837 | 7.82×10^-55^ | 3.08×10^-53^ | down | XP_013792812.1 | *Limulus polyphemus* |
| Forkhead | TRINITY_DN33657_c0_g4 | 4.87845 | 17.46728 | -1.84016 | 3.65×10^-31^ | 8.15×10^-30^ | down | XP_016843963.1 | *Nasonia vitripennis* |
| Forkhead | TRINITY_DN34476_c0_g3 | 5.401466 | 10.84873 | -1.0061 | 1.55×10^-27^ | 3.06×10^-26^ | down | KDR22393.1 | *Zootermopsis nevadensis* |
| Kazal-type serine protease inhibitor domain | TRINITY_DN36353_c0_g1 | 2.580911 | 8.172572 | -1.66291 | 7.87×10^-17^ | 1.01×10^-15^ | down | XP_011677925.1 | *Strongylocentrotus purpuratus* |
| Piwi | TRINITY_DN37569_c2_g2 | 42.23031 | 94.31813 | -1.15926 | 4.97×10^-266^ | 1.17×10^-263^ | down | AGV15456.1 | *Portunus trituberculatus* |
| Piwi | TRINITY_DN33432_c1_g2 | 5.60739 | 20.55359 | -1.87399 | 5.84×10^-48^ | 2.01×10^-46^ | down | ADK25181.1 | *Litopenaeus vannamei* |

Mro-IRi: Mro-IRi group; GFPi: GFP RNAi group.

Supplementary table 3 Significantly differential expression of molt, reproduction and sex-related candidate genes in the comparative transcriptomic analysis of premature ovary in Mro-IR knockdown

| **Gene Description** | **Gene ID** | **MeanTPM**  **(Mro-IRi)** | **MeanTPM**  **(GFPi)** | **log2Fold**  **Change** | **pValue** | **qValue** | **Result** | **GenBank No.** | **Species** |
| --- | --- | --- | --- | --- | --- | --- | --- | --- | --- |
| Calcium binding and coiled-coil domain | TRINITY_DN40165_c1_g3 | 44.71297 | 18.00137 | 1.312587 | 1.62×10^-13^ | 7.15×10^-12^ | up | - | *-* |
| Crustacean CHH/MIH/GIH neurohormone | TRINITY_DN38201_c0_g1 | 109.9181 | 269.3245 | -1.29292 | 1.41×10^-67^ | 4.08×10^-65^ | down | AAL40916.1 | *Macrobrachium rosenbergii* |
| Cytochrome b5 | TRINITY_DN34710_c1_g3 | 12.08496 | 4.549205 | 1.409526 | 9.02×10^-32^ | 1.07×10^-29^ | up | XP_011444831.1 | *Crassostrea gigas* |
| Cytochrome b6 | TRINITY_DN32576_c1_g5 | 7.03415 | 1.702959 | 2.046332 | 1.38×10^-17^ | 8.18×10^-16^ | up | AEE62517.1 | *Dendroctonus ponderosae* |
| Iron-containing alcohol dehydrogenase | TRINITY_DN32042_c0_g6 | 5.379568 | 2.559247 | 1.071771 | 5.40×10^-7^ | 1.10×10^-5^ | up | XP_004078811.1 | *Oryzias latipes* |
| Ion channel | TRINITY_DN36762_c2_g1 | 331.0995 | 1609.88 | -2.28162 | 1.99×10^-302^ | 3.75×10^-299^ | down | XP_013771695.1 | *Limulus polyphemus* |
| Ion channel | TRINITY_DN31465_c0_g2 | 5.738121 | 2.79868 | 1.035832 | 4.12×10^-12^ | 1.59×10^-10^ | up | XP_013780582.1 | *Limulus polyphemus* |
| Ligand-gated ion channel （Ionotropic Glutamate Receptor ） | TRINITY_DN31700_c3_g1 | 9.094173 | 3.038704 | 1.581486 | 9.30×10^-28^ | 9.51×10^-26^ | up | XP_016766363.1 | *Apis mellifera* |
| Ligand-gated ion channel | TRINITY_DN37617_c0_g1 | 4.862179 | 10.06575 | -1.04978 | 5.03×10^-20^ | 3.44×10^-18^ | down | - | *-* |
| Ligand-gated ion channel | TRINITY_DN39839_c1_g4 | 6.928069 | 14.76844 | -1.09199 | 6.35×10^-13^ | 2.65×10^-11^ | down | - | *-* |
| Ligand-gated ion channel （Ionotropic Glutamate Receptor ） | TRINITY_DN41056_c7_g1 | 3.960461 | 9.796631 | -1.30662 | 4.42×10^-4^ | 4.71×10^-3^ | down | CEF34378.1 | *Coenobita clypeatus* |
| Ligand-gated ion channel （Ionotropic Glutamate Receptor ） | TRINITY_DN34507_c0_g3 | 43.69292 | 118.571 | -1.44028 | 4.53×10^-39^ | 6.61×10^-37^ | down | CEF34377.1 | *Coenobita clypeatus* |
| Ligand-gated ion channel （Ionotropic Glutamate Receptor ） | TRINITY_DN41072_c5_g1 | 5.024644 | 2.38456 | 1.075298 | 1.20×10^-3^ | 1.10×10^-2^ | up | - | *-* |
| Lipocalin （intracellular fatty acid binding protein ） | TRINITY_DN31034_c0_g5 | 151.8469 | 30.33868 | 2.323388 | 4.31×10^-188^ | 4.39×10^-185^ | up | ABE77153.1 | *Pacifastacus leniusculus* |
| Lipocalin （apolipoprotein D ） | TRINITY_DN32777_c0_g1 | 17.35308 | 7.72711 | 1.167191 | 1.70×10^-10^ | 5.52×10^-9^ | up | XP_516965.1 | *Pan troglodytes* |
| Sodium/calcium exchanger protein | TRINITY_DN33729_c1_g2 | 4.515651 | 9.385672 | -1.05553 | 3.69×10^-5^ | 5.24×10^-4^ | down | KZS06718.1 | *Daphnia magna* |
| Cytochrome P450 CYP315a1 | TRINITY_DN36782_c0_g5 | 4.84574 | 14.92398 | -1.62284 | 7.17×10^-47^ | 1.31×10^-44^ | down | AJF94636.1 | *Portunus trituberculatus* |
| Cytochrome P450 CYP2 | TRINITY_DN34569_c5_g1 | 3.642367 | 15.67013 | -2.10507 | 8.98×10^-12^ | 3.34×10^-10^ | down | ALA09303.1 | *Eriocheir sinensis* |
| Cytochrome P450 2G1 | TRINITY_DN33517_c1_g2 | 8.49845 | 0.0001 | 16.37491 | 6.14×10^-11^ | 2.08×10^-9^ | up | XP_006006256.1 | *Latimeria chalumnae* |
| Cytochrome P450 CYP2 | TRINITY_DN34706_c2_g6 | 2.84513 | 6.52173 | -1.19676 | 2.05×10^-3^ | 1.72×10^-2^ | down | AHZ97878.1 | *Portunus trituberculatus* |
| Phosphoenolpyruvate carboxykinase | TRINITY_DN32940_c1_g1 | 32.19137 | 206.3063 | -2.68004 | 1.20×10^-306^ | 2.48×10^-303^ | down | CAB85964.1 | *Litopenaeus vannamei* |
| Phosphoenolpyruvate carboxykinase | TRINITY_DN39916_c2_g2 | 23.1541 | 118.094 | -2.3506 | 1.64×10^-185^ | 1.59×10^-182^ | down | ALK82313.1 | *Macrobrachium nipponense* |
| Phosphoenolpyruvate carboxykinase | TRINITY_DN39916_c2_g4 | 66.98178 | 159.8627 | -1.25499 | 1.71×10^-84^ | 6.60×10^-82^ | down | ALK82313.1 | *Macrobrachium nipponense* |
| Ion channel regulatory protein UNC-93 | TRINITY_DN35807_c1_g2 | 5.655197 | 2.74009 | 1.045354 | 1.86×10^-4^ | 2.22×10^-3^ | up | XP_969831.1 | *Tribolium castaneum* |
| Ankyrin repeats ( NF-kappa B inhibitor alpha ) | TRINITY_DN36427_c1_g4 | 10.62293 | 22.62568 | -1.09078 | 4.03×10^-136^ | 2.87×10^-133^ | down | AET34918.1 | *Macrobrachium rosenbergii* |
| Ankyrin repeats | TRINITY_DN32819_c1_g5 | 11.58697 | 5.340927 | 1.117342 | 2.86×10^-20^ | 1.98×10^-18^ | up | KDR06504.1 | *Zootermopsis nevadensis* |
| Ankyrin repeats (E3 ubiquitin-protein ligase) | TRINITY_DN39383_c1_g2 | 8.67962 | 3.563745 | 1.284238 | 3.42×10^-5^ | 4.91×10^-4^ | up | XP_013774252.1 | *Limulus polyphemus* |
| autophagy-related protein | TRINITY_DN39975_c1_g1 | 252.9291 | 10.65485 | 4.569151 | 4.85×10^-15^ | 2.40×10^-13^ | up | XP_015584958.1 | *Cephus cinctus* |
| E3 ubiquitin-protein ligase | TRINITY_DN31459_c2_g4 | 5.84449 | 2.272689 | 1.362677 | 6.01×10^-5^ | 8.16×10^-4^ | up | XP_013774853.1 | *Limulus polyphemus* |
| Fatty acid hydroxylase | TRINITY_DN39611_c1_g1 | 15.21502 | 3.515523 | 2.113685 | 2.61×10^-70^ | 8.08×10^-68^ | up | KRT81761.1 | *Oryctes borbonicus* |
| Hsp70 | TRINITY_DN37281_c0_g3 | 0.246617 | 6.162023 | -4.64306 | 1.98×10^-3^ | 1.66×10^-2^ | down | AFQ62791.1 | *Litopenaeus vannamei* |
| low-density lipoprotein receptor | TRINITY_DN40799_c1_g1 | 151.1835 | 37.4695 | 2.012512 | 0 | 0 | up | XP_014044934.1 | *Salmo salar* |
| Male sterility protein | TRINITY_DN32264_c4_g3 | 37.8145 | 15.43049 | 1.293156 | 1.76×10^-57^ | 4.10×10^-55^ | up | XP_002429200.1 | *Pediculus humanus corporis* |
| Peptidase (signal peptide peptidase) | TRINITY_DN34400_c0_g1 | 84.73841 | 36.13625 | 1.229569 | 7.02×10^-7^ | 1.41×10^-5^ | up | XP_015910089.1 | *Parasteatoda tepidariorum* |
| Peptidase (cathepsin C ) | TRINITY_DN38629_c0_g6 | 56.80663 | 25.05385 | 1.181027 | 1.03×10^-69^ | 3.14×10^-67^ | up | ACK57788.1 | *Litopenaeus vannamei* |
| Peptidase (matrix metalloproteinase) | TRINITY_DN36915_c0_g1 | 6.4599 | 1.030323 | 2.648415 | 4.56×10^-49^ | 8.79×10^-47^ | up | XP_013782452.1 | *Limulus polyphemus* |
| Peptidase (membrane metallo-endopeptidase) | TRINITY_DN34954_c2_g1 | 17.88773 | 3.622749 | 2.303814 | 3.14×10^-49^ | 6.09×10^-47^ | up | XP_015173767.1 | *Polistes dominula* |
| Phosphoglucomutase/phosphomannomutase | TRINITY_DN37524_c1_g4 | 34.45274 | 8.38938 | 2.037983 | 1.04×10^-161^ | 9.17×10^-159^ | up | XP_015586696.1 | *Cephus cinctus* |
| Trypsin | TRINITY_DN33589_c0_g1 | 14.47231 | 0.352003 | 5.361565 | 8.60×10^-27^ | 8.43×10^-25^ | up | AAL04113.2 | *Homarus americanus* |
| Trypsin | TRINITY_DN34622_c3_g4 | 3.710216 | 11.1012 | -1.58114 | 3.04×10^-22^ | 2.37×10^-20^ | down | ACY66494.3 | *Scylla paramamosain* |
| Trypsin | TRINITY_DN32093_c0_g2 | 1.82587 | 9.069096 | -2.31237 | 8.17×10^-17^ | 4.58×10^-15^ | down | ABG67960.1 | *Callinectes sapidus* |
| Trypsin | TRINITY_DN39665_c1_g1 | 0.649481 | 5.211963 | -3.00447 | 1.64×10^-3^ | 1.42×10^-2^ | down | AAL04113.2 | *Homarus americanus* |
| Forkhead box L2 | TRINITY_DN35254_c1_g3 | 14.1871 | 0.179013 | 6.308372 | 4.09×10^-163^ | 3.66×10^-160^ | up | ALD48735.1 | *Procambarus clarkii* |

Mro-IRi: Mro-IRi group; GFPi: GFP RNAi group.
